# Chemical investigation on the cultures of the fungus *Xylaria carpophila*

**DOI:** 10.1007/s13659-011-0011-y

**Published:** 2011-09-27

**Authors:** Xia Yin, Tao Feng, Zheng-Hui Li, Jia Su, Yan Li, Ning-Hua Tan, Ji-Kai Liu

**Affiliations:** 1 State Key Laboratory of Phytochemistry and Plant Resources in West China, Kunming Institute of Botany, Chinese Academy of Sciences, Kunming, 650201 China; 2 Graduate University of Chinese Academy of Sciences, Beijing, 100039 China

**Keywords:** *Xylaria carpophila*, sesquiterpenes, cyclopeptide, cytotoxicity

## Abstract

A chemical investigation on the cultures of *Xylaria carpophila* led to the isolation of one known cyclopeptide cyclo(Nmethyl-_l_-Phe-_l_-Pro-_l_-Leu-_d_-Ile-_l_-Val) (**1**), five new sesquiterpenes, named as xylcarpins A–E (**2**–**6**), and another known compound (**7**). The structures were determined by extensive NMR and MS spectroscopic analysis. The absolute configuration of **1** was established by use of Marfey’s method and ROESY spectroscopic data. All compounds were tested for their cytotoxicities against five human cancer cell lines. Compound **7** showed week inhibitory activity.

**Electronic Supplementary Material:**

Supplementary material is available for this article at 10.1007/s13659-011-0011-y and is accessible for authorized users.

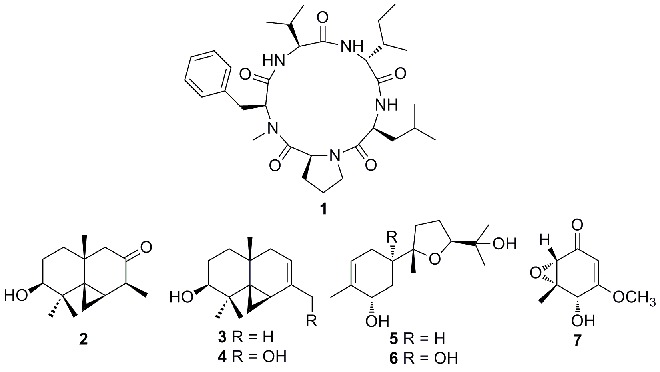

## Introduction

Fungi of the genus *Xylaria* are known to produce many types of bioactive compounds. Previous studies revealed that isopimarane diterpene glycosides,[1] xylopimarane,[2] 4,6,8(14),22-tetraen-3-one,[3] xylarisin,[4] coloratin A,[5] kolokoside A,[6] cytochalasins,[7] clonostachydiol,[7] xylobovide,[7] 07H239-A,[8] cytochalasin D,[9] multiplolides A and B,[10] sordaricin,[11] mellisol,[12] 1,8-dihydroxynaphthol 1-*O*-*α*-glucopyranoside,[12] benzoquinones,[13] and integric acid[14] from some species of this genus showed diverse pharmacological properties, including cytotoxic, antimicrobial, antifungal, anthelmintic, antivirus, and antimalarial activities. But the chemical constituents of *Xylaria carpophila* have received little attention. Our current investigation on the cultures of *X. carpophila* led to the isolation of one known cyclopeptide, cyclo(N-methyl-L-Phe-L-Pro-L-Leu-D-Ile-L-Val) (**1**), five new sesquiterpenes, named as xylcarpins A–E (**2**–**6**), and one known compound (**7**). The structures of cyclo(N-methyl-L-Phe-L-Pro-L-Leu-D-Ile-L-Val) (**1**), and xylcarpins A–E (**2**–**6**) were elucidated by means of spectroscopic methods. The known compound (**7**) was identified as (4*S*,5*S*,6*S*)-5,6-epoxy-4-hydroxy-3-methoxy-5-methyl-cyclohex-2-en-1-one[15] by comparison with data in the literature. All of these compounds were tested for their cytotoxicities against five human cancer cell lines.

## Results and Discussion

Compound **1** was obtained as a white powder. The molecular formula was established as $C_{32}H_{49}N_5O_5$ on the basis of its HRESIMS at *m/z* 584.3802 $[M + H]^+$ (calcd for $C_{32}H_{50}N_5O_5$, 584.3811) indicating 11 degrees of unsaturation.

**1**

**2**

**3** R = H
**4** R = OH

**5** R = H
**6** R = OH

**7**

The IR absorption bands at 3292, 1642 $cm^{-1}$ indicated the presence of NH and CO groups. The 1D NMR spectroscopic data (Table 1) revealed the presence of three NH protons ($\delta_H$ 9.76, 9.29, and 7.70, respectively), six methyl groups as well

*To whom correspondence should be addressed. E-mail: jkliu@mail.kib.ac.cn as one amide *N*-$CH_3$ signal ($\delta_H$ 3.47; $\delta_c$ 31.6), six methylene signals, eight $sp^3$ methine signals, a monosubstituted benzene ring signals, and five carbonyl carbons. Preliminary analysis of these NMR and MS data suggested that compound **1** might be a pentapeptide. In the $^1H$-$^1H$ COSY spectrum (Figure 1), six fragments were established indicating that compound **1** comprised one leucine, one isoleucine, one valine, one proline and one phenylalanine. The HMBC correlation of $\delta_H$ 3.47 (3H, s, Me-*N*) with $\delta_C$ 58.2 (CH, *α*-carbon of the phenylalanine) suggested the methyl substitution at *N*-atom of phenylalane. In addition, the HMBC correlations also established the amino acids sequence to be N-methyl-Phe-Pro-Leu-Ile-Val (Figure 1). These data indicated that compound **1** was structurally the same as cyclo(N-methyl-L-Phe-L-Val-D-Ile-L-Leu-L-Pro).[16]

Advanced Marfey's method was applied to identify the configuration of the amino acid, and showed the presence of L-Leu as well as L-Val. The configurations of *N*-MePhe[1], Pro[2], Leu[3], Ile[4] and Val[5] were determined to be L, L, L, D, L respectively by the application of the Marfey's method, the analysis of ROESY correlations (Table 1 and Figure 1) and the comparison with the data of cyclo(N-methyl-L-Phe-L-Pro-L-Leu-D-Ile-L-Val).[16]

**Figure 1.** Key 2D NMR correlations for **1**.

Compound **2** was purified as a white powder. The molecular formula was determined as $C_{15}H_{24}O_2$ by HRESIMS at *m/z* 259.1671 $[M + Na]^+$ (calcd for $C_{15}H_{24}O_2Na$, 259.1673), with four degrees of unsaturation. The IR spectrum indicated the presence of hydroxy (3441 $cm^{-1}$) and carbonyl (1706 $cm^{-1}$)

**Table 1. NMR data of 1 in pyridine-*d*$_5$.**

| amino acid | position | $\delta_C$ (multi) | $\delta_H$ (*J* in Hz) | HMBC | ROESY |
|---|---|---|---|---|---|
| N-methyl Phe | CO | 172.0, qC | | *α*, NH-Val | |
| | *α* | 58.2, CH | 5.49, m | *β*, *γ*, N-$CH_3$ | *β*, NHVal, *α*Val |
| | *β* | 36.0, $CH_2$ | 3.23, dd (14.2, 9.1); 3.37, dd (14.2, 7.7) | *α*, ortho, *γ* | *α*, ortho |
| | *γ* | 138.3, qC | | meta | |
| | ortho | 129.8, CH | 7.23, m | meta, para | *α*, *β*, *β*Pro |
| | meta | 129.3, CH | 7.28, m | para | *γ*Pro, *β* |
| | para | 127.0, CH | 7.25, m | ortho | |
| | N-$CH_3$ | 31.6 | 3.47, s | *α* | *α*Pro, *β*Pro |
| Pro | CO | 173.4, qC | | *α*, *β*, N-Me-Phe | |
| | *α* | 60.3, CH | 5.59, m | *β*, *γ* | *β*, N-Me, *α*Leu[3], |
| | *β* | 31.5, $CH_2$ | 1.45, m; 2.03, m | *γ*, *δ* | *γ* |
| | *γ* | 21.9, $CH_2$ | 1.66, m | *β* | *β*, *δ* |
| | *δ* | 47.0, $CH_2$ | 3.73, m; 3.82, m | *β*, *γ* | *γ* |
| Leu | CO | 170.2, qC | | *α* | |
| | *α* | 48.5, CH | 5.40, m | *β*, *γ* | *β*, *α*Pro |
| | *β* | 42.7, $CH_2$ | 1.96, m | *α*, *γ*, *δ* | *α* |
| | *γ* | 25.7, CH | 1.79, m | *α*, *β*, *δ* | *α*, *β* |
| | *δ* | 23.6, $CH_3$; 23.2, $CH_3$ | 0.95, d (6.6); 0.99, d (6.4) | *β*, *γ* | |
| | NH | | 9.76, d (9.3) | *α* | *α*, *β*, *α*Leu[3] |
| Ile | CO | 172.2, qC | | *α*, NH-L-Leu | |
| | *α* | 57.4, CH | 4.90, m | *β*, *δ*, *γ* | *β*, *γ*, NHLeu[3] |
| | *β* | 39.6, CH | 1.99, m | *α*, *δ*, *γ* | *α* |
| | *γ* | 27.5, $CH_2$ | 1.29, m; 1.67, m | *δ* | *α* |
| | *β*-$CH_3$ | 12.4, $CH_3$ | 0.90, t (7.4) | | |
| | *δ* | 15.3, $CH_3$ | 1.05, d (6.8) | | |
| | NH | | 7.70, d (8.7) | *α*, | *α*, *α*Val, |
| Val | CO | 172.6, qC | | *α*, NH-D-Leu | |
| | *α* | 59.4, CH | 4.56, t (10.1) | *β*, *γ* | *β*, NH, *α*N-MePhe, NHLeu[4] |
| | *β* | 27.7, CH | 2.27, m | *α*, *γ* | *α* |
| | *γ* | 19.4, $CH_3$; 20.8, $CH_3$ | 0.86, d (6.9); 1.03, d (6.6) | | |
| | NH | | 9.29, d (9.7) | *α* | *α*, *β*, *α*N-MePhe |

**Table 2.** $^{1}H$ **and** $^{13}C$ **NMR data of 2–4 in** $CDCl_3$**.**

| | 2 | | 3 | | 4 | |
|---|---|---|---|---|---|---|
| Entry | $\delta_H$ (*J* in Hz) | $\delta_C$ mult. | $\delta_H$ (*J* in Hz) | $\delta_C$ mult. | $\delta_H$ (*J* in Hz) | $\delta_C$ mult. |
| 1 | 2.61, m | 40.2, CH | | 135.5, qC | | 139.3, qC |
| 2 | | 214.5, qC | 5.02 br. d (6.4) | 114.6, CH | 5.30 br. d (6.1) | 116.5, CH |
| 3 | 2.13, d (16.8); 1.77, d (16.8) | 52.3, $CH_2$ | 1.76, m; 1.44, m | 40.8, $CH_2$ | 1.83, m; 1.54, m | 40.3, $CH_2$ |
| 4 | | 37.6, qC | | 31.3, qC | | 31.6, qC |
| 5 | 1.45, m | 36.6, $CH_2$ | 1.54, m | 34.5, $CH_2$ | 1.51, m | 34.4, $CH_2$ |
| | | | 1.15, m | | 1.18, m | |
| 6 | 1.79, m | 28.2, $CH_2$ | 1.72, m | 28.6, $CH_2$ | 1.80, m | 28.5, $CH_2$ |
| | | | 1.78, m | | 1.73, m | |
| 7 | 3.41, ddd (10.8, 5.3, 5.3) | 78.4, CH | 3.34, ddd (11.5, 5.6, 5.2) | 78.5, CH | 3.34, ddd (11.7, 5.4, 5.2) | 78.4, CH |
| 8 | | 39.5, qC | | 39.5, qC | | 39.5, qC |
| 9 | | 32.5, qC | | 34.8, qC | | 34.5, qC |
| 10 | 1.34, m | 23.0, CH | 1.24, m | 22.1, CH | 1.44, m | 18.8, CH |
| 11 | 1.16, d (8.0) | 17.2, $CH_3$ | 1.80, s | 23.6, $CH_3$ | 4.13, d (4.8) | 67.1, $CH_2$ |
| 12 | 0.56, m | 6.2, $CH_2$ | 0.72, m | 10.7, $CH_2$ | 0.79, m | 10.9, $CH_2$ |
| | 0.49, m | | 0.71, m | | 0.74, m | |
| 13 | 0.76, s | 24.9, $CH_3$ | 0.75, s | 24.3, $CH_3$ | 0.77, s | 24.3, $CH_3$ |
| 14 | 1.09, s | 21.1, $CH_3$ | 1.09, s | 19.3, $CH_3$ | 1.10, s | 20.9, $CH_3$ |
| 15 | 1.15, s | 28.3, $CH_3$ | 1.12, s | 28.6, $CH_3$ | 1.15, s | 28.6, $CH_3$ |
| 7-OH | 1.40, d (5.3) | | 1.30, d (5.6) | | 1.33, d (5.2) | |
| 11-OH | | | | | 1.34, t (4.8) | |

groups. The $^{1}H$ NMR spectrum (Table 2) showed signals for an oxymethine proton at $\delta_H$ 3.41, three singlet methyl signals ($\delta_H$ 0.76, 1.09, 1.15) and one doublet methyl signal ($\delta_H$ 1.16, $J$ = 8.0 Hz). The $^{13}C$ NMR and DEPT spectra (Table 2) showed 15 carbon resonances ascribable for four methyls, four methylenes, three methines (including one oxygen-bearing methine), and four quaternary carbons (one of which is a carbonyl carbon). Apart from one degree of unsaturation occupied by one carbonyl group, the remaining three degrees of unsaturation indicated that compound **2** should possess a three-ring system including one three-membered carbon ring according to the typical methylene signal at $\delta_C$ 6.2. In the $^{1}H$-$^{1}H$ COSY spectrum, two fragments was established as shown with bold line in Figure 2. These data suggested that compound **2** possessed a backbone related to the known compound thujopsene.[17] The major differences including a hydroxy group at C-3 ($\delta_C$ 78.4) and a carbonyl group at C-2

**Figure 2.** Key 2D NMR correlations for **2**.

($\delta_C$ 214.5) were established by the HMBC and $^{1}H$-$^{1}H$ COSY spectra analysis (Figure 2). The relative configuration of **2** was elucidated by the ROESY experiment (Figure 2). The ROESY correlations of H-13 with H-7 and H-10, and H-10 with H-1 implied that OH-7, $CH_3$-14, three-membered ring and $CH_3$-11 to be the same side. ROESY correlations of $H_a$-3 ($\delta_H$ 2.13) with $H_b$-12 (0.49) and H-15 suggested that the $CH_3$-15 was at the same side with the three-membered ring. Therefore, the structure of **2** was established and named as xylcarpin A.

Compound **3** was isolated as a white powder with a molecular formula of $C_{15}H_{24}O$ based on the positive ion HRESIMS at $m/z$ 203.1800 $[M - OH]^+$ (calcd for $C_{15}H_{23}$, 203.1799). The NMR data (Table 2) were similar to those of **2** with the major differences being that the signal of the carbonyl carbon at C-2 was absent, showing instead a pair of double bond functionality between C-1 ($\delta_C$ 135.5) and C-2 ($\delta_C$ 114.6), which was supported by the HMBC correlations from $\delta_H$ 1.80 (3H, s, H-11) to $\delta_C$ 135.5 (s, C-1) and from $\delta_H$ 1.76 (1H, m, H-3) to $\delta_C$ 114.6 (d, C-2). The same ROESY correlations of **3** and **2** indicated their identical relative configuration. Therefore, compound **3** was established and named as xylcarpin B.

Compound **4** was obtained as a white powder. The IR spectrum showed the existence of hydroxy groups (3417 $cm^{-1}$). The NMR data were quite similar to those of **3**, except for the methyl at C-11 was oxygenated into a methylene ($\delta_C$ 67.1), which was supported by the HMBC correlation of $\delta_H$ 4.13 (2H, d, $J$ = 4.8 Hz, H-11) with $\delta_C$ 139.3 (s, C-1). Furthermore, the same ROESY data of **3** and **4** suggested the same relative configuration of them. Therefore, compound **4** was established as depicted, and named as xylcarpin C.

Compound **5** was isolated as a colorless oil with the molecular formula of $C_{15}H_{26}O_3$ based on the HRESIMS at $m/z$ 277.1773 $[M + Na]^+$ (calcd for $C_{15}H_{26}O_3Na$, 277.1779). IR absorption bands at 3424 $cm^{-1}$ indicated the presence of hydroxy groups. The $^{13}C$ NMR spectrum (Table 3) showed 15 carbons including one trisubstituted double-bond resonance at $\delta_C$ 134.6 and 125.5 and four oxygenated carbons at $\delta_C$ 86.3, 84.6, 70.6 and 68.8. Four methyl signals at $\delta_C$ 28.0, 24.2, 24.1 and 21.1 were also observed. Except for the location of one hydroxy group , the NMR spectroscopic data features of **5**

**Table 3. $^1H$ and $^{13}C$ NMR data of 5 and 6 in $CDCl_3$.**

| | 5 | | 6 | |
|---|---|---|---|---|
| Entry | $\delta_H$ (*J* in Hz) | $\delta_C$ mult. | $\delta_H$ (*J* in Hz) | $\delta_C$ mult. |
| 1 | 2.05, m | 27.4, $CH_2$ | 2.30, m | 34.2, $CH_2$ |
| | 1.76, m | | 1.99, m | |
| 2 | 5.55, m | 125.5, CH | 5.26, br. s | 121.1, CH |
| 3 | | 134.6, qC | | 137.6, qC |
| 4 | 4.01, br. s | 68.8, CH | 4.22, m | 68.4, CH |
| 5 | 1.98, m | 33.2, $CH_2$ | 1.96, m | 40.0, $CH_2$ |
| | 1.43, m | | 1.40, m | |
| 6 | 1.83, m | 37.8, CH | | 70.0, qC |
| 7 | | 84.6, qC | | 89.0, qC |
| 8 | 1.87, m | 35.7, $CH_2$ | 2.31, m | 33.7, $CH_2$ |
| | 1.61, m | | 1.53, m | |
| 9 | 1.83, m | 26.6, $CH_2$ | 1.95, m | 27.2, $CH_2$ |
| | 1.77, m | | 1.85, m | |
| 10 | 3.67, dd | 86.3, CH | 3.79, dd | 85.6, CH |
| | (10.0, 5.5) | | (7.5, 7.4) | |
| 11 | | 70.6, qC | | 71.9, qC |
| 12 | 1.18, s | 28.0, $CH_3$ | 1.21, s | 28.0, $CH_3$ |
| 13 | 1.09, s | 24.2, $CH_3$ | 1.05, s | 26.5, $CH_3$ |
| 14 | 1.12, s | 24.1, $CH_3$ | 1.15, s | 23.5, $CH_3$ |
| 15 | 1.77, s | 21.1, $CH_3$ | 1.70, s | 19.5, $CH_3$ |
| 4-OH | | | 3.58, d (7.1) | |
| 6-OH | | | 3.70, s | |
| 11-OH | | | 3.75, s | |

were quite similar to those of (1*R*)-1-hydroxybisabololoxide B.[18] The hydroxy group was attached to C-4 as supported by the HMBC correlation from $\delta_H$ 1.77 (3H, s, H-15) to $\delta_C$ 68.8 (d, C-4). By comparison of the NMR spectroscopic data and the optical rotation data of (1*R*)-1-hydroxybisabololoxide B,[18] the relative configurations of C-6, C-7 and C-10 in **5** were deduced as same as those in (1*R*)-1-hydroxybisabololoxide B. In the ROESY spectrum, the correlation between H-4 and H-6 was not observed, indicating that the hydroxy group at C-4 should be $\alpha$-oriented, which was also supported by the broad singlet of H-4. Compound **5** was established and named as xylcarpin D.

Compound **6** was obtained as colorless oil. The molecular formula of $C_{15}H_{26}O_4$ was indicated by the HRESIMS at *m/z* 293.1724 $[M + Na]^+$ (calcd for $C_{15}H_{26}O_4Na$, 293.1728). 1D NMR data were very similar to those of compound **5** except for an oxygenated quaternary carbon at $\delta_C$ 70.0 (s, C-6) in **6** instead of a methine in **5**. Analysis of the HMBC spectrum suggested that one hydroxy group was placed at C-6 by the correlation of $\delta_C$ 2.30 (1H, m, H-1) and 1.96 (1H, m, H-5) with $\delta_C$ 70.0 (s, C-6). Detailed analysis of other 2D NMR data suggested that other parts of **6** were identical to those of **5**. Therefore, compound **6** was established and named as xylcarpin E.

Compounds **1**–**7** were evaluated for their cytotoxicities against five hunman cancer cell lines using the MTT method as reported previously[19]. Compound **7** showed week cytotoxicity against four of the tested cell lines (Table 4), while other compounds were inactive to all tested cell lines ($IC_{50}$ values of more than 40 $\mu$M).

**Table 4. Cytotoxicity for 7 ($IC_{50}$, $\mu$M).**

| Entry | HL-60 | SMMC-7721 | A-549 | MCF-7 | SW480 |
|---|---|---|---|---|---|
| **7** | 23.1 | > 40 | 35.7 | 28.5 | 29.0 |
| cisplatin[a] | 1.3 | 15.4 | 17.3 | 15.8 | 24.4 |

[a]positive control.

## Experimental Section

**General Experimental Procedures**. Optical rotations (OR) were recorded on a Jasco P-1020 digital polarimeter. Infrared spectroscopy (IR) spectra were obtained on a Bruker Tensor 27 FT-IR spectrometer with KBr pellets. Nuclear Magnetic Resonance (NMR) spectra were obtained on a Bruker Avance III 600 MHz spectrometer with tetramethylsilane (TMS) as an internal standard at room temperature. Electrospray ionization-mass spectra (ESI-MS) and high-resolution (HR) ESI-MS were recorded on a VG Autospec-3000 mass spectrometer and an API QSTAR Pulsar I spectrometer. Silica gel (200–300 mesh, Qingdao Marine Chemical Ltd., China) and Sephadex LH-20 (Amersham Biosciences, Sweden) were used for open column chromatography (CC). MPLC was performed on a Büchi Sepacore system (Büchi Labortechnik AG, Switzerland), and columns packed with RP-18 silica gel (40–75 $\mu$m, Fuji Silysia Chemical Ltd., Japan). Preparative HPLC was performed on an Agilent 1100 liquid chromatography system equipped with a Zorbax SB-C18 column (9.4 mm × 150 mm). Fractions were monitored by TLC. Spots were visualized by heating silica gel plates immersed in Vanillin-$H_2SO_4$ in ethanol.

**Fungal Material and Cultivation Conditions.** The fungus *Xylaria carpophila* (Pers.) Fr. was collected from Gaoligong Mountains in Yunnan Province, China, in 2008. The fungus was identified by Prof. Zhu-Liang Yang at the Kunming Institute of Botany. A voucher specimen was deposited at the Herbarium of Kunming Institute of Botany, Chinese Academy of Sciences. The culture medium consisted of glucose (5%), peptone from porcine meat (0.15%), yeast powder (0.5%), $KH_2PO_4$ (0.5%) and MgSO4. Fermentation was carried out on a shaker at 160RPM for 25 days.

**Extraction and Isolation.** The culture broth (21 L) was filtered, and the filtrate was extracted three times with EtOAc while the mycelium was extracted three times with $CH_3Cl$-MeOH (1:1). The EtOAc layer together with the mycelium extraction was concentrated under reduced pressure to give a crude extract (4.5 g), and this residue was subjected to CC over silica gel (200–300 mesh) eluted with a gradient of $CH_3Cl$-MeOH (1:0 → 0:1) to obtain eight fractions (1–8). Fraction 2 (570 mg) applied to MPLC (MeOH-$H_2O$, eluting from 50:50 to 100:0 for 100 mins with a flow of 20 mL/min) then isolated and purified by repeated silica gel chromatography, eluted with petroleum ether/EtOAc (2:1), followed by Sephadex LH-20 ($Me_2CO$) to afford **2** (2.5 mg), **3** (0.7 mg), **5** (1.4 mg) and **7** (1.0 mg). Compound **1** (6 mg) precipitated from fraction 2. Fraction 3 (130 mg) was applied to MPLC (MeOH-$H_2O$, eluting from 0:1 to 1:0 for 60 mins with a flow rate of 20 mL/min) to give four subfractions (3a-3d). Fraction 3d was subjected to silica gel, eluted with $CH_3Cl/Me_2CO$ (10:1) to yield compound **4** (3 mg). Fraction 5 was isolated by preparative HPLC (MeCN-$H_2O$, eluting from 0:100 to 50:50 for

50 mins followed by 50:50 to 100:0 for 30mins with a flow rate of 20 mL/min) to give a mixture, and compound **6** (2 mg) was separated from the mixtures by Sephadex LH-20 ($Me_2CO$).

**Cyclo(N-methyl-L-Phe-L-Pro-L-Leu-D-Ile-L-Val) (1):** white powder; $[\alpha]_D^{22}$ − 59.5 (*c* 0.26, MeOH); IR (KBr) $\nu_{max}$ 3292, 2960，1642, 1527, 1383, 740, 698 $cm^{-1}$; $^1H$ (600 MHz) and $^{13}C$ NMR (150 MHz) data (methanol-$d_4$), see Table 1; positive ion HRESIMS *m/z* 584.3802 $[M + H]^+$ (calcd for $C_{32}H_{50}N_5O_5$, 584.3811).

**Xylcarpin A (2):** white powder; $[\alpha]_D^{22}$ + 161.6 (*c* 0.19, $Me_2CO$); IR (KBr) $\nu_{max}$ 3441, 2923, 1706, 1630 $cm^{-1}$; $^1H$ (600 MHz) and $^{13}C$ NMR (150 MHz) data ($CDCl_3$), see Table 2; positive ion HRESIMS *m/z* 259.1671 $[M + Na]^+$ (calcd for $C_{15}H_{24}O_2Na$, 259.1673).

**Xylcarpin B (3):** white powder; $[\alpha]_D^{22}$ + 12.5 (*c* 0.11, $Me_2CO$); IR (KBr) $\nu_{max}$ 3420, 2958, 2924, 2855, 1630, 1453, 1066, 1042 $cm^{-1}$; $^1H$ (600 MHz) and $^{13}C$ NMR (150 MHz) data ($CDCl_3$), see Table 2; positive ion HRESIMS *m/z* 203.1800 $[M - OH]^+$ (calcd for $C_{15}H_{23}$, 203.1799).

**Xylcarpin C (4):** white powder; $[\alpha]_D^{22}$ + 86.3 (*c* 0.10, $Me_2CO$); IR (KBr) $\nu_{max}$ 3417, 2937, 2920, 1631, 1456, , 1064, 1044, 1006 $cm^{-1}$; $^1H$ (600 MHz) and $^{13}C$ NMR (150 MHz) data ($CDCl_3$), see Table 2; positive ion HRESIMS *m/z* 259.1667 $[M + Na]^+$ (calcd for $C_{15}H_{26}O_2Na$, 259.1673).

**Xylcarpin D (5):** colorless oil; $[\alpha]_D^{22}$ − 64.0 (*c* 0.14, $Me_2CO$); IR (KBr) $\nu_{max}$ 3424, 2970, 2933, 2876, 1640, 1452, 1375, 1161, 1127, 1080, 1058, 1033, 957, 942, 807 $cm^{-1}$; $^1H$ (600 MHz) and $^{13}C$ NMR (150 MHz) data ($CDCl_3$), see Table 3; positive ion HRESIMS *m/z* 277.1773 $[M + Na]^+$ (calcd for $C_{15}H_{26}O_3$, 277.1779).

**Xylcarpin E (6):** colorless oil; $[\alpha]_D^{22}$ − 36.6 (*c* 0.12, $Me_2CO$); IR (KBr) $\nu_{max}$ 3430, 2969, 2924, 2854, 1633, 1455, 1378, 1079, 1057, 1037 $cm^{-1}$; $^1H$ (600 MHz) and $^{13}C$ NMR (150 MHz) data ($CDCl_3$), see Table 3; positive ion HRESIMS *m/z* 293.1724 $[M + Na]^+$ (calcd for $C_{15}H_{26}O_4Na$, 293.1728).

**Marfey's Reaction to Identify the Absolute Configuration of 1.** Compound **1** (1.0 mg) was dissolved in 1 mL of 6 N HCl and heated at 120°C for 20 h. After that, the hydrolyzate must be evaporated to dryness under a stream of $N_2$ to remove traces of HCl. The hydrolyzate was added a 1% solution of L-FDDA (100 $\mu$L) in acetone and 1M $NaHCO_3$ (20 $\mu$L), and the mixture was heated at 40℃ for 45 min. The standard amino acid L-Val and D-/L-Leu were treated the same way, and all the reactions must be stopped by addition of HCl (2 M; 10 $\mu$L).[16,20] When all these sovents were evaporated, The N-[(dinitrophenyl)-5-L-alanine amide] amino acid derivatives were redissolved in MeCN-$H_2O$ (1:1) followed by HPLC analysis (Agilent 1100 liquid chromatography system equipped with a Zorbax SB-C18 column (4.6 mm × 150 mm); solvents: (A) water + 0.05% TFA, (B) MeCN; linear gradient: 0 min 10%, 40min 50%, 1 mL/min). HPLC analysis of the reaction products of the hydrolysate of compound **1** showed the presence of both L-Leu as well as L-Val.

**Cytotoxicity Assay.** Human myeloid leukemia HL-60, hepatocellular carcinoma SMMC-7721, lung cancer A-549 cells, breast cancer MCF-7 and colon cancer SW480 cell lines were used in the cytoxic assay. All the cells were cultured in RPMI-1640 or DMEM medium (Hyclone, USA), supplemented with 10% fetal bovine serum (Hyclone, USA) in 5% $CO_2$ at 37 °C. The cytotoxicity assay was performed according to the MTT (3-(4,5-dimethylthiazol-2-yl)-2,5-diphenyl tetrazolium bromide) method in 96-well microplates.[19] Briefly, 100 $\mu$L adherent cells were seeded into each well of 96-well cell culture plates and allowed to adhere for 12 h before drug addition, while suspended cells were seeded just before drug addition with initial density of $1 \times 10^5$ cells/mL. Each tumor cell line was exposed to the test compound dissolved in DMSO at concentrations of 0.0625, 0.32, 1.6, 8, and 40 $\mu$M in triplicates for 48 h, with cisplatin (Sigma, USA) as a positive control. After compound treatment, cell viability was detected and a cell growth curve was graphed. $IC_{50}$ values were calculated by Reed and Muench's method.[21]

## Electronic Supplementary Material

Supplementary material is available in the online version of this article at http://dx.doi.org/10.1007/s13659-011-0011-y and is accessible for authorized users.

## Acknowledgments

This project was supported by the National Basic Research Program of China (973 Program, 2009CB522300), the National Natural Sciences Foundation of China (30830113), and MOST (2009ZX09501-029).

**Open Access** This article is distributed under the terms of the Creative Commons Attribution License which permits any use, distribution, and reproduction in any medium, provided the original author(s) and source are credited.

## Electronic supplementary material

Supplementary material, approximately 2.39 MB.
