# Supplementary material for: Chemical investigation on the cultures of the fungus Xylaria carpophila
Source: Nat Prod Bioprospect. 2011 Sep 27;1(2):75–80. doi: 10.1007/s13659-011-0011-y (PMC4131652; doi:10.1007/s13659-011-0011-y)

**Electronic Supplementary Material**

# Chemical investigation on the cultures of the fungus *Xylaria carpophila*

Xia YIN,[a,b] Tao FENG,[a] Zheng-Hui LI,[a] Jia SU,[a] Yan LI,[a] Ning-Hua TAN,[a] and Ji-Kai LIU[a,]*

[a]State Key Laboratory of Phytochemistry and Plant Resources in West China, Kunming Institute of Botany, Chinese Academy of Sciences, Kunming 650201, China

[b]Graduate University of Chinese Academy of Sciences, Beijing 100039, China

Received 5 August 2011; Accepted 20 September 2011

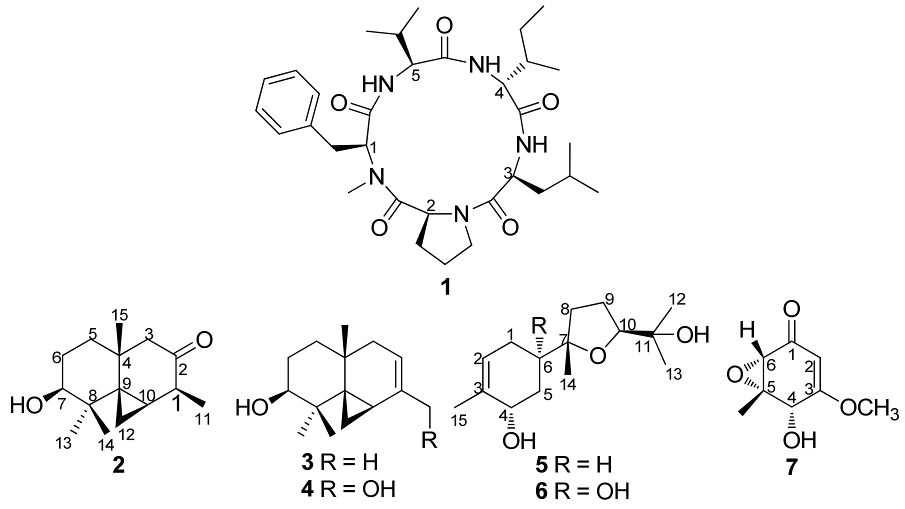


Structures of compounds **1**–**7**.

*To whom correspondence should be addressed. E-mail: jkliu@mail.kib.ac.cn.

## Table of Contents

**Figure 1S-8S.** NMR, MS spectra and Marfey´s method reports of compound **1**

**Figure 9S-15S.** NMR and MS spectra of compound **2**

**Figure 16S-22S.** NMR and MS spectra of compound **3**

**Figure 23S-29S.** NMR and MS spectra of compound **4**

**Figure 30S-36S.** NMR and MS spectra of compound **5**

**Figure 37S-41S.** NMR and MS spectra of compound **6**

**Figure 42S-43S.** $^{1}$H NMR and $^{13}$C NMR spectra of compound **7**

Figure 1S. $^{1}$H NMR of compound **1**.

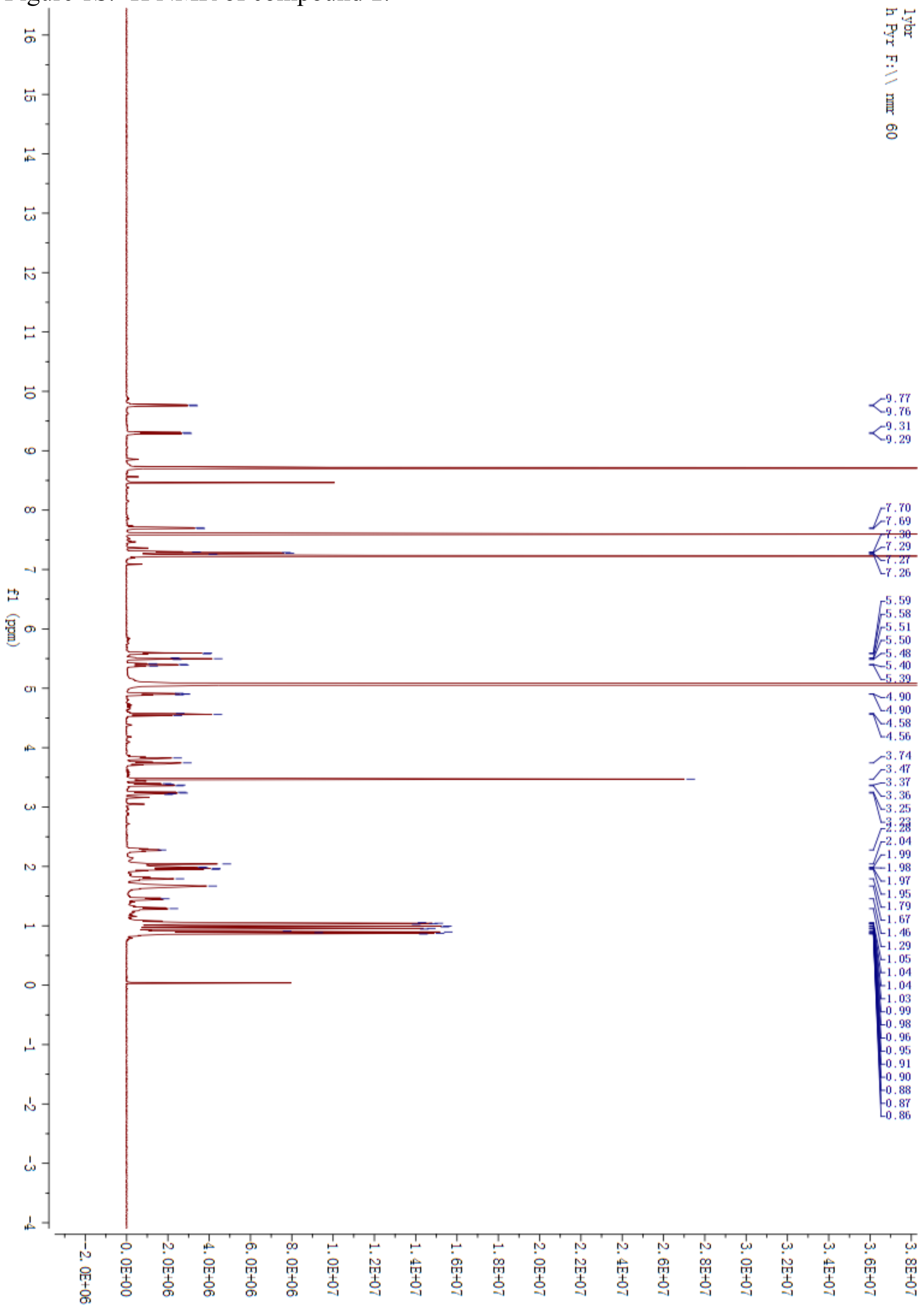

Figure 2S. $^{13}$C NMR and DEPT of compound **1**

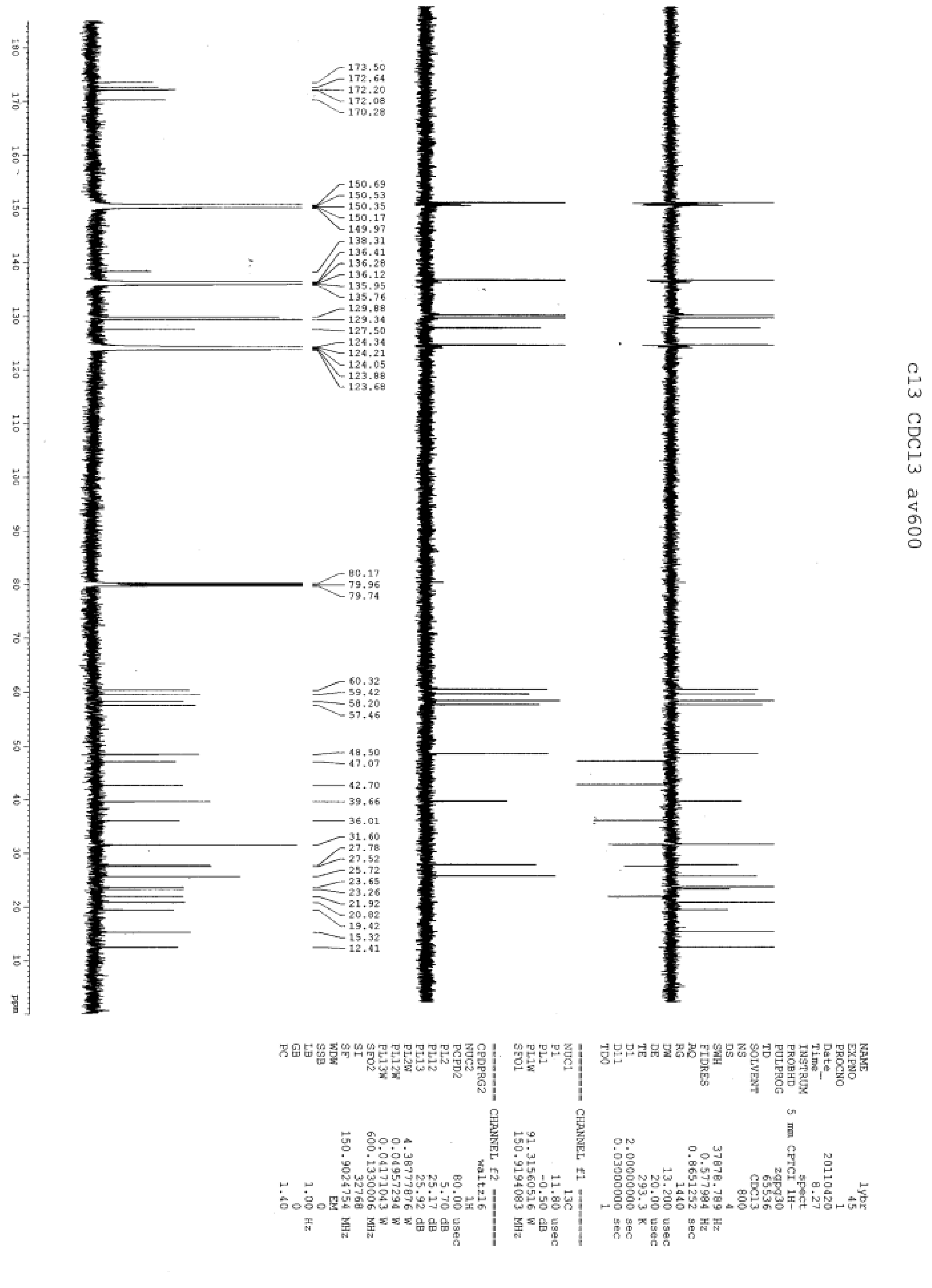

Figure 3S. HSQC of compound **1**

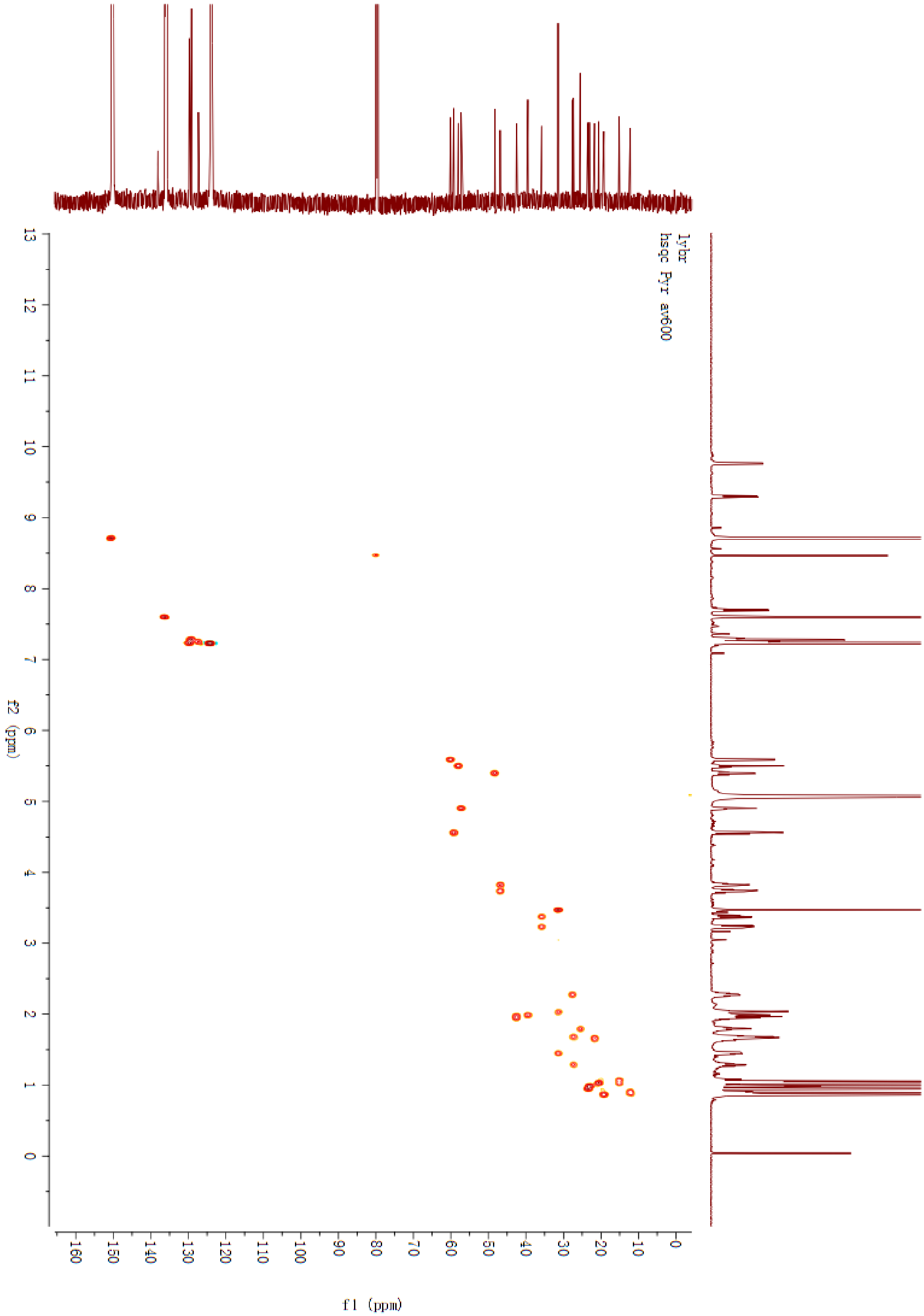

Figure 4S. HMBC of compound **1**

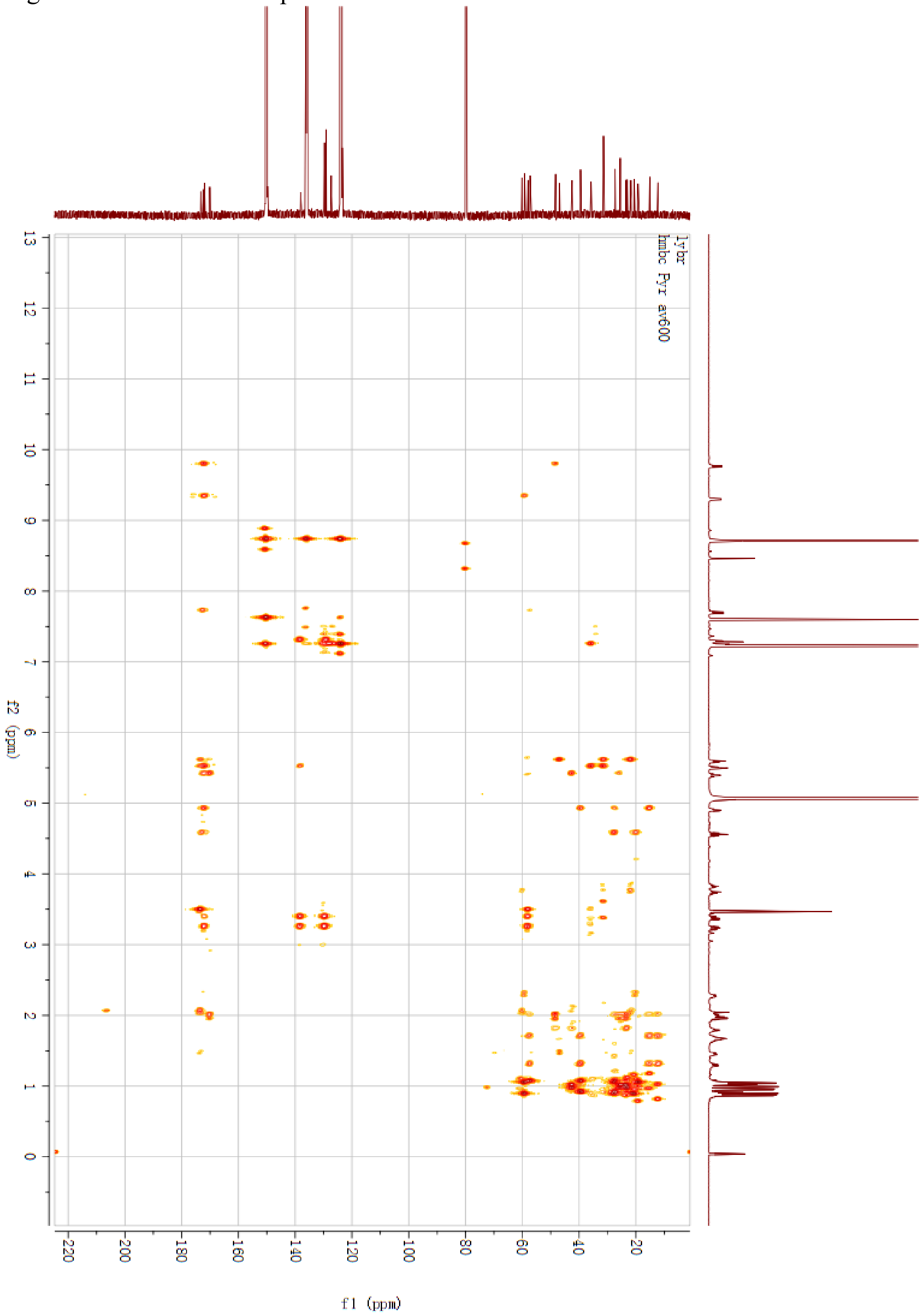

Figure 5S. $^1$H-$^1$H COSY of compound **1**.

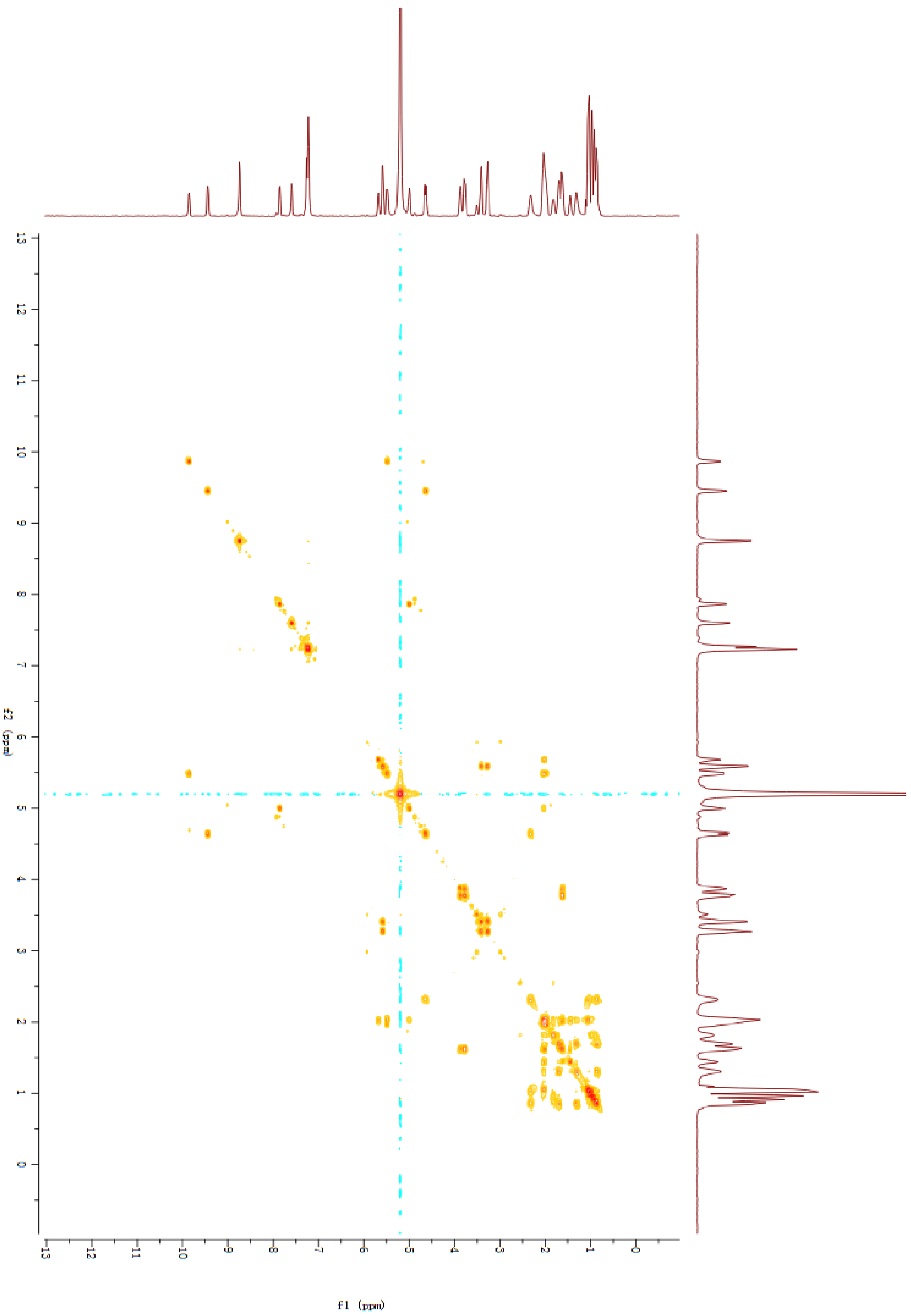

Figure 6S. ROESY of compound **1**.

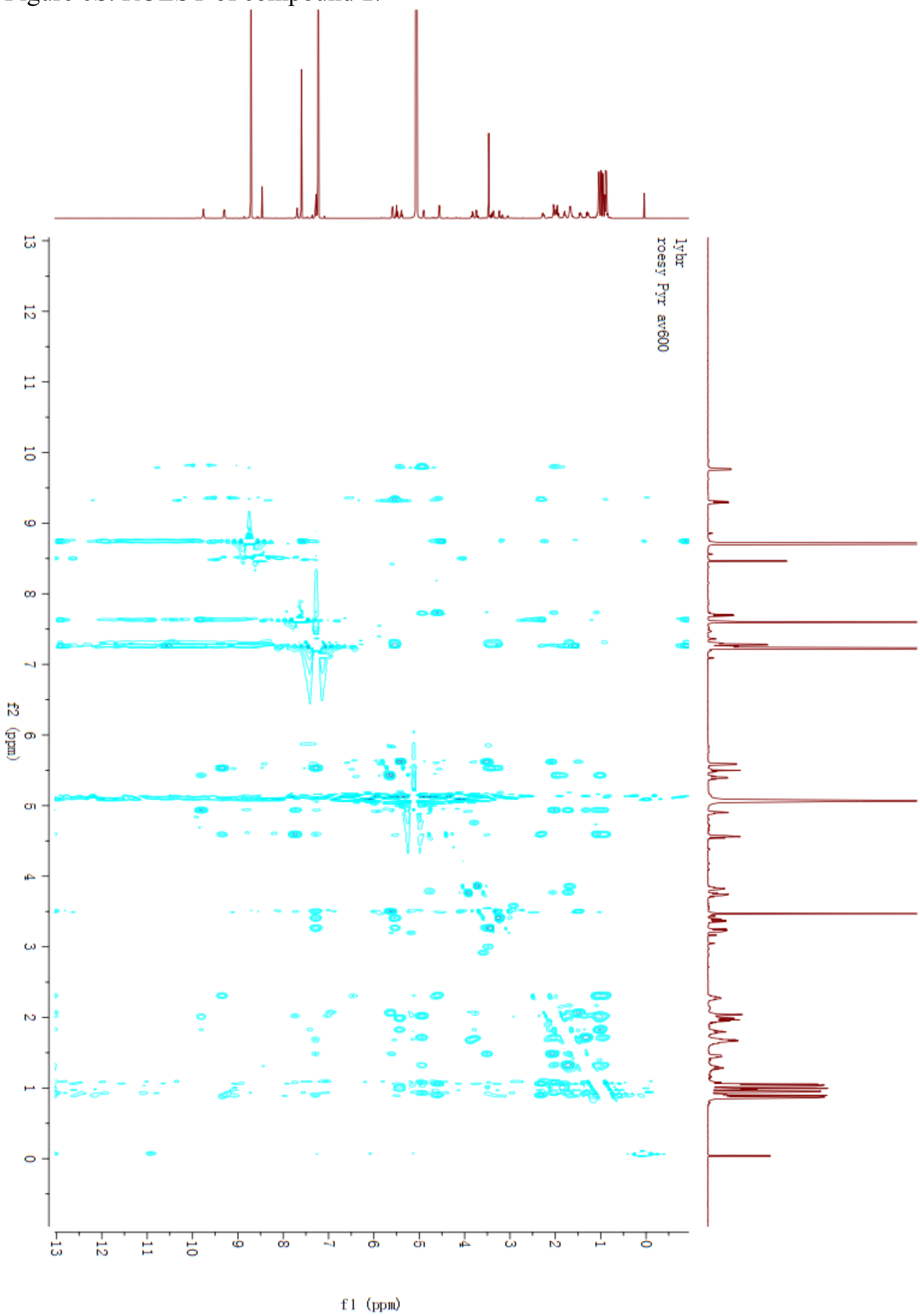

Figure 7S. HRESIMS of compound **1**.

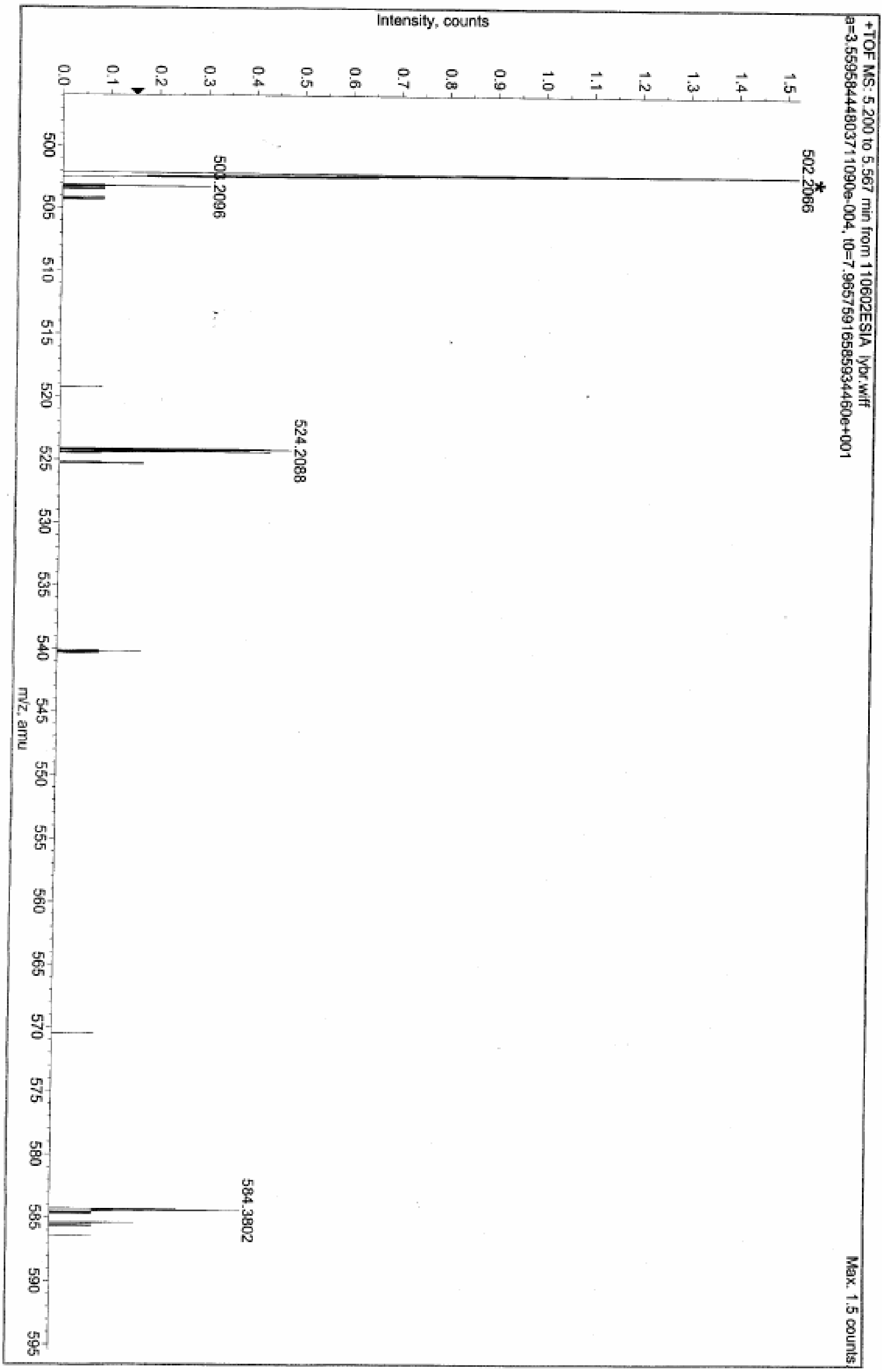

Figure 8S. Reports of Marfey´s method of compound **1**.

D,L-Leu

L32.327min

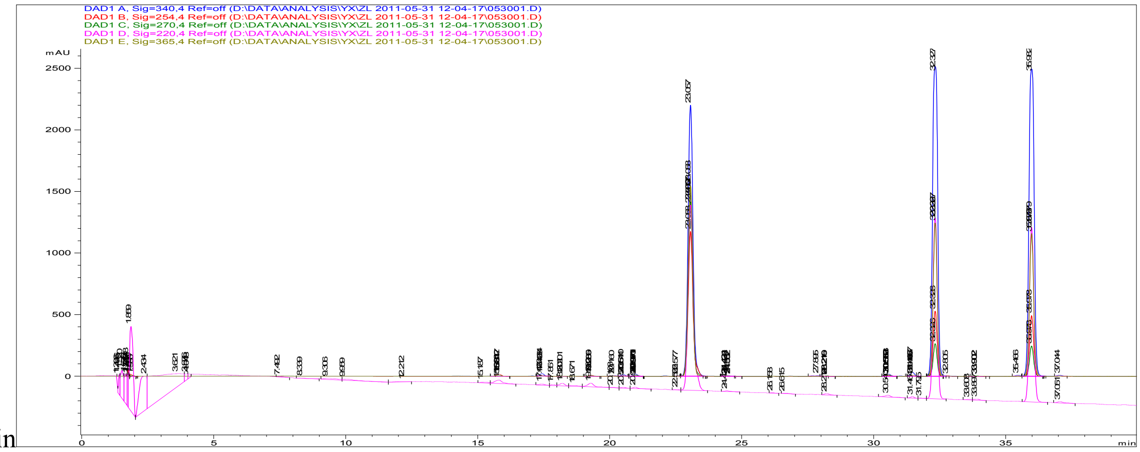


D35.982min

L-Val 27.887min

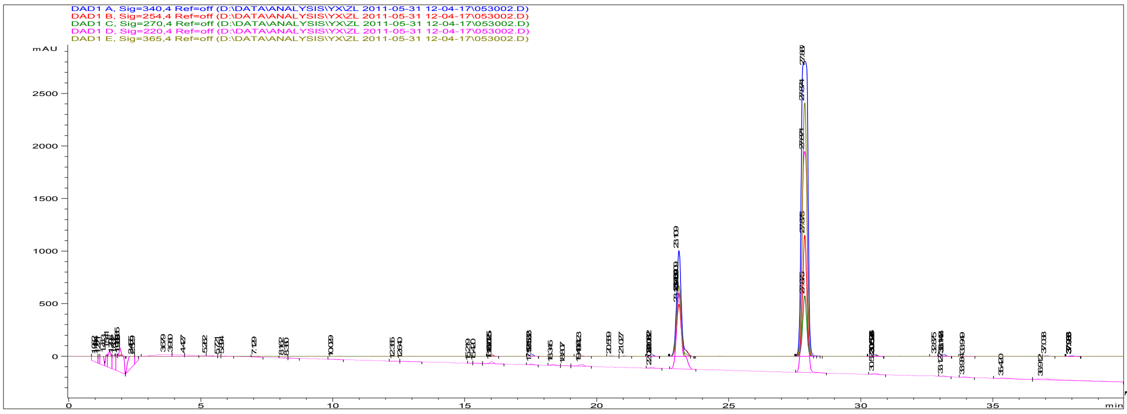


The hydrolyzate of compound **1**.

L-Val 27.812min

L-Leu 32.312min

D-Leu 35.535min

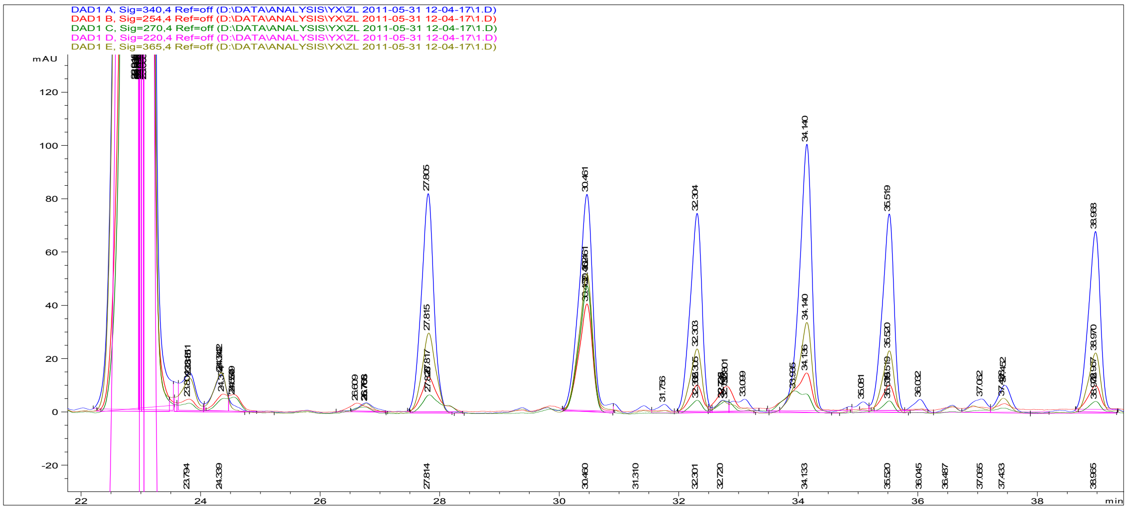


Figure 9S. $^{1}$H NMR of compound **2**.

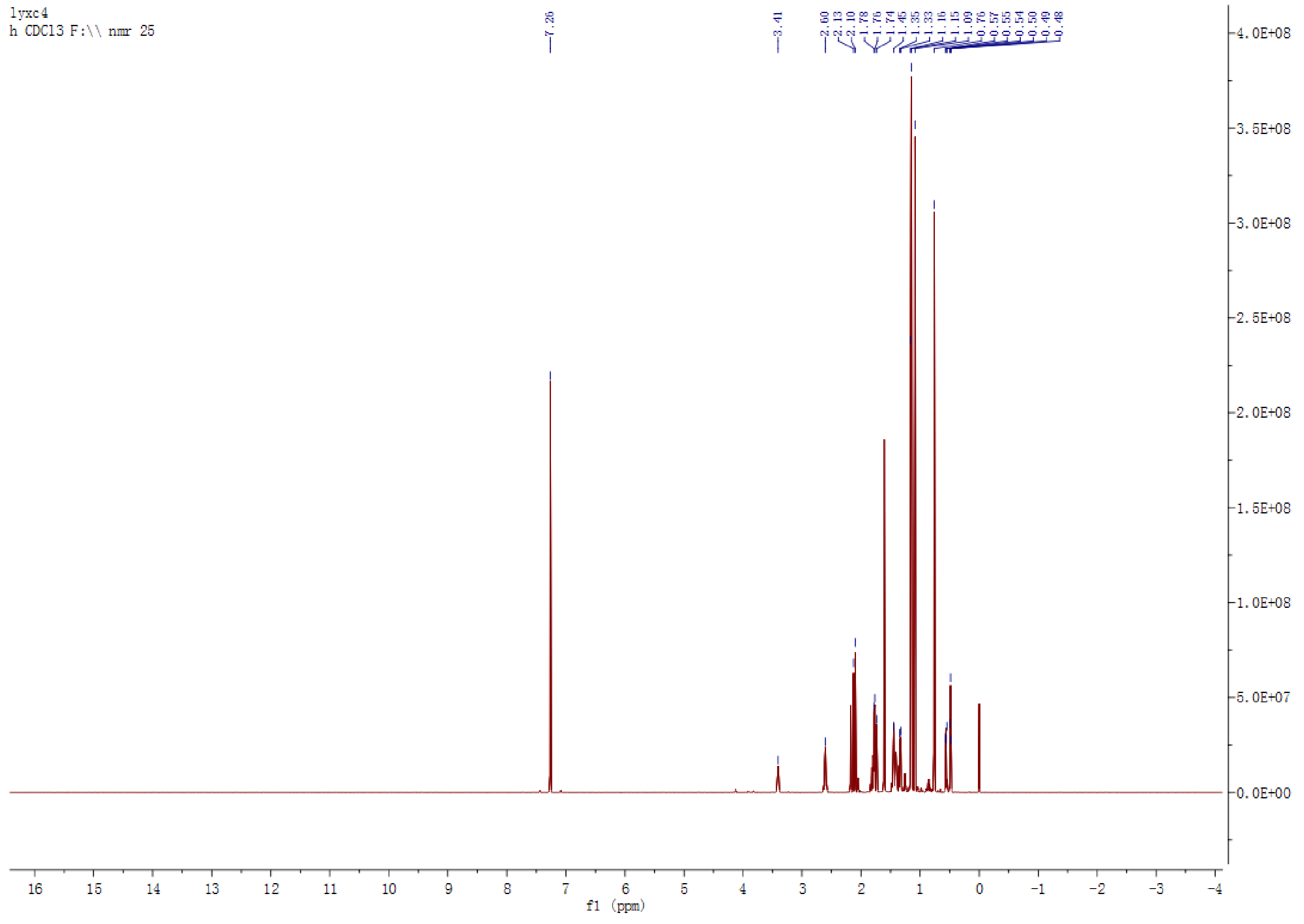
lyxc4
h CDCl3 F:\\ nmr 25
7.26
3.41
2.60
2.13
2.10
1.78
1.76
1.74
1.45
1.35
1.33
1.16
1.15
1.09
0.76
0.57
0.55
0.54
0.50
0.49
0.48
4.0E+08
3.5E+08
3.0E+08
2.5E+08
2.0E+08
1.5E+08
1.0E+08
5.0E+07
0.0E+00
16 15 14 13 12 11 10 9 8 7 6 5 4 3 2 1 0 -1 -2 -3 -4
f1 (ppm)

Figure 10S. $^{13}$C NMR and DEPT of compound **2**.

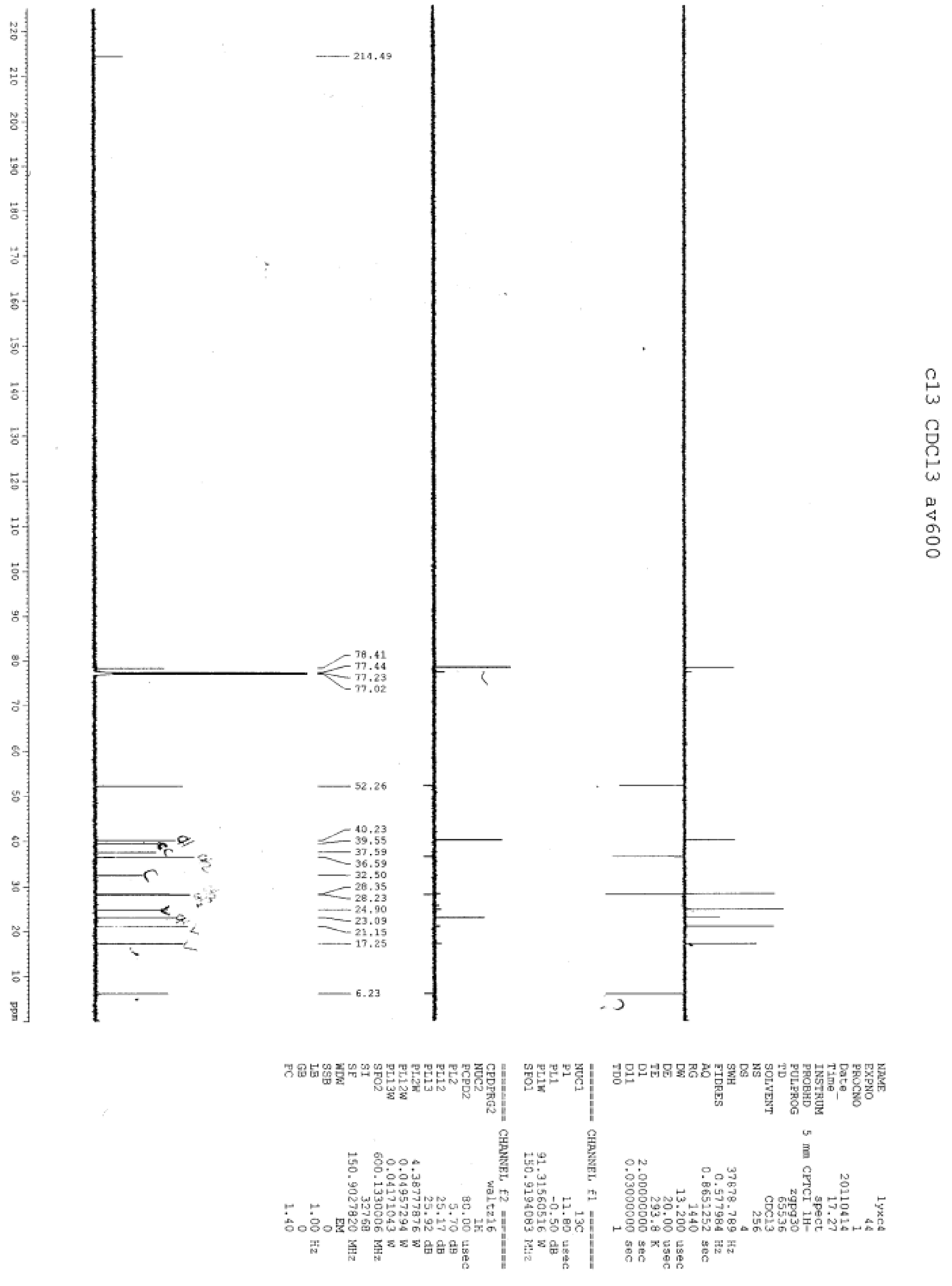

Figure 11S. HSQC of compound **2**.

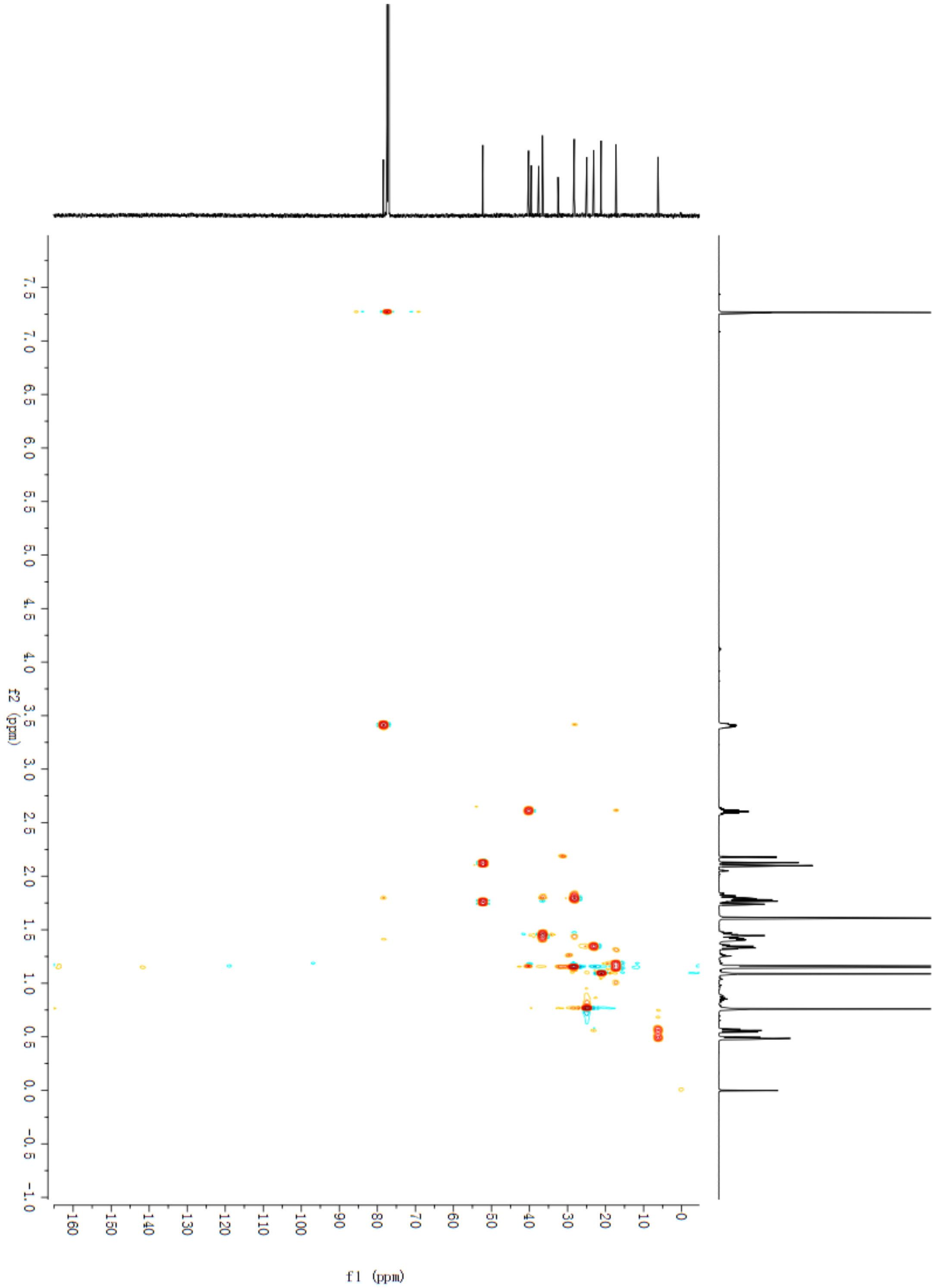

Figure 12S. HMBC of compound **2**.

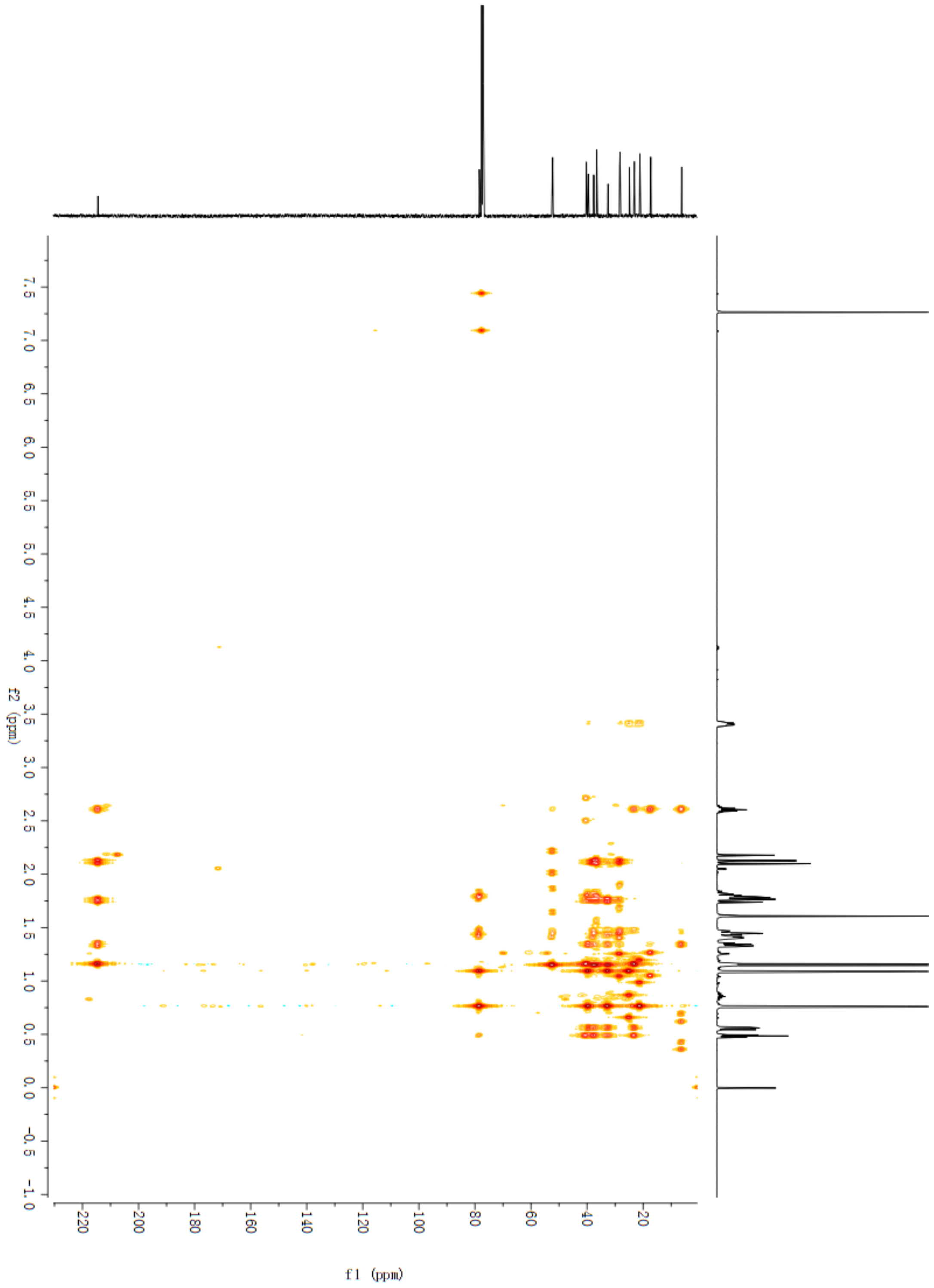

Figure 13S. $^{1}H$-$^{1}H$ COSY of compound **2**

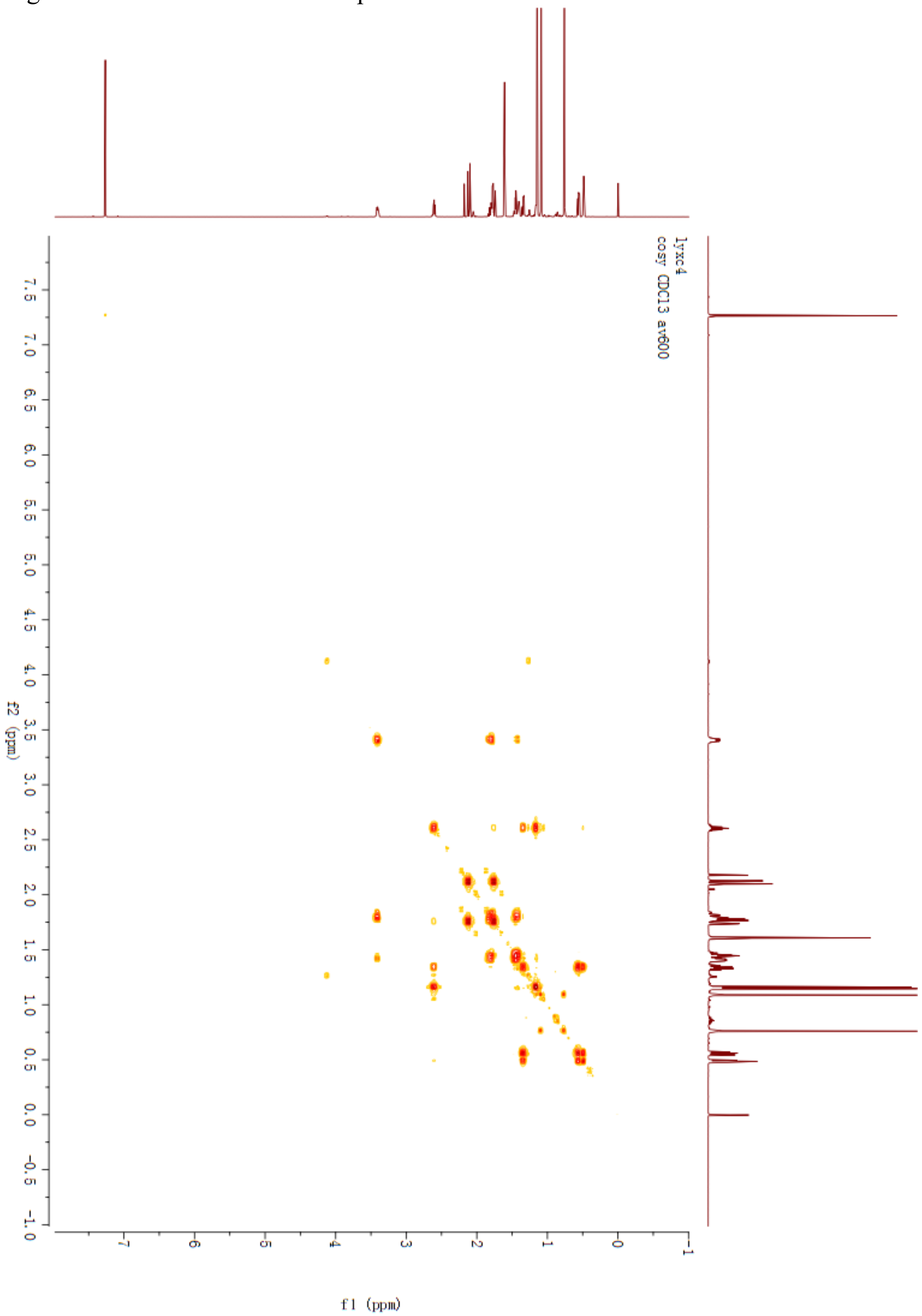

Figure 14S. ROESY of compound **2**.

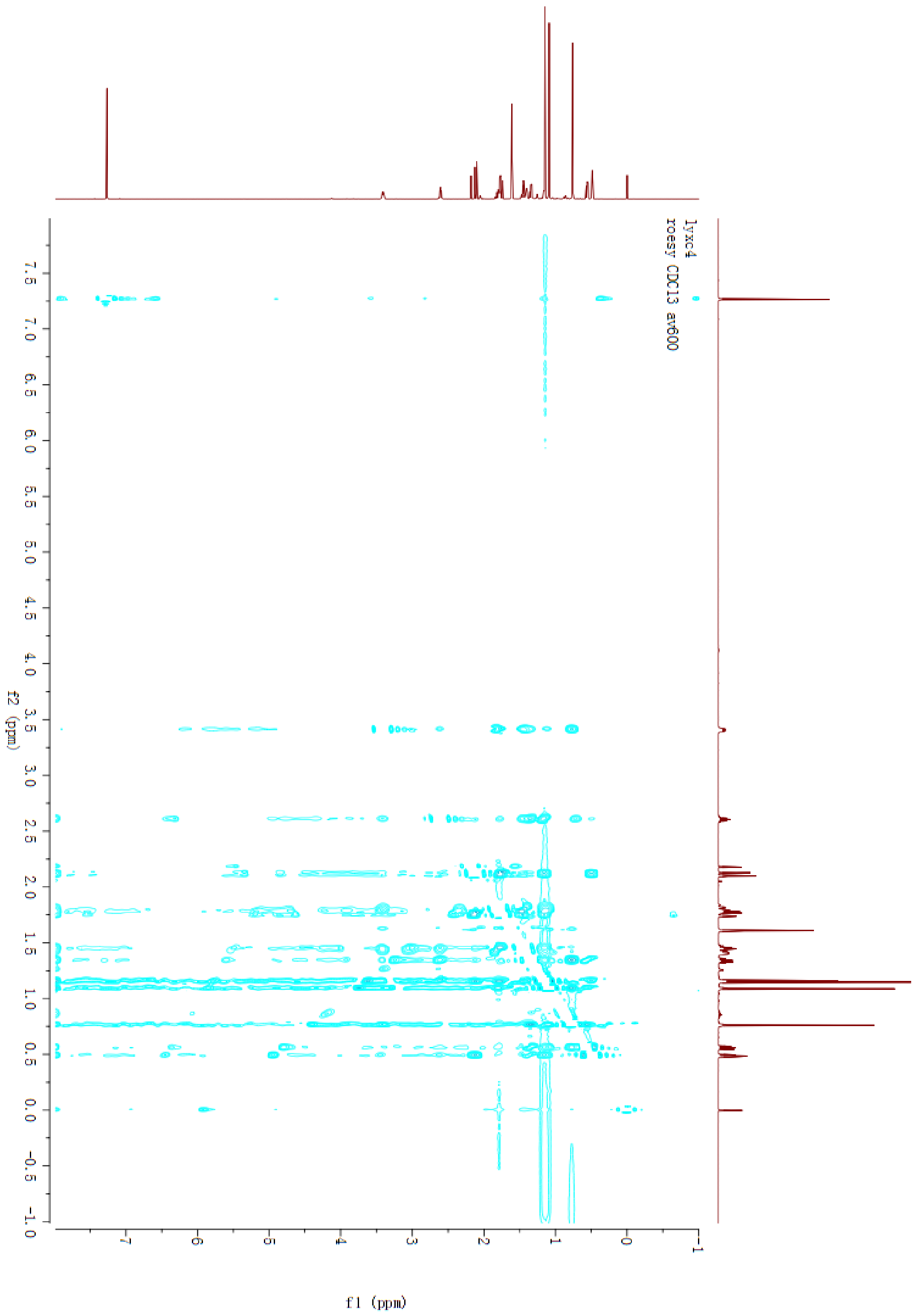

Figure 15S. HRESIMS of compound **2**.

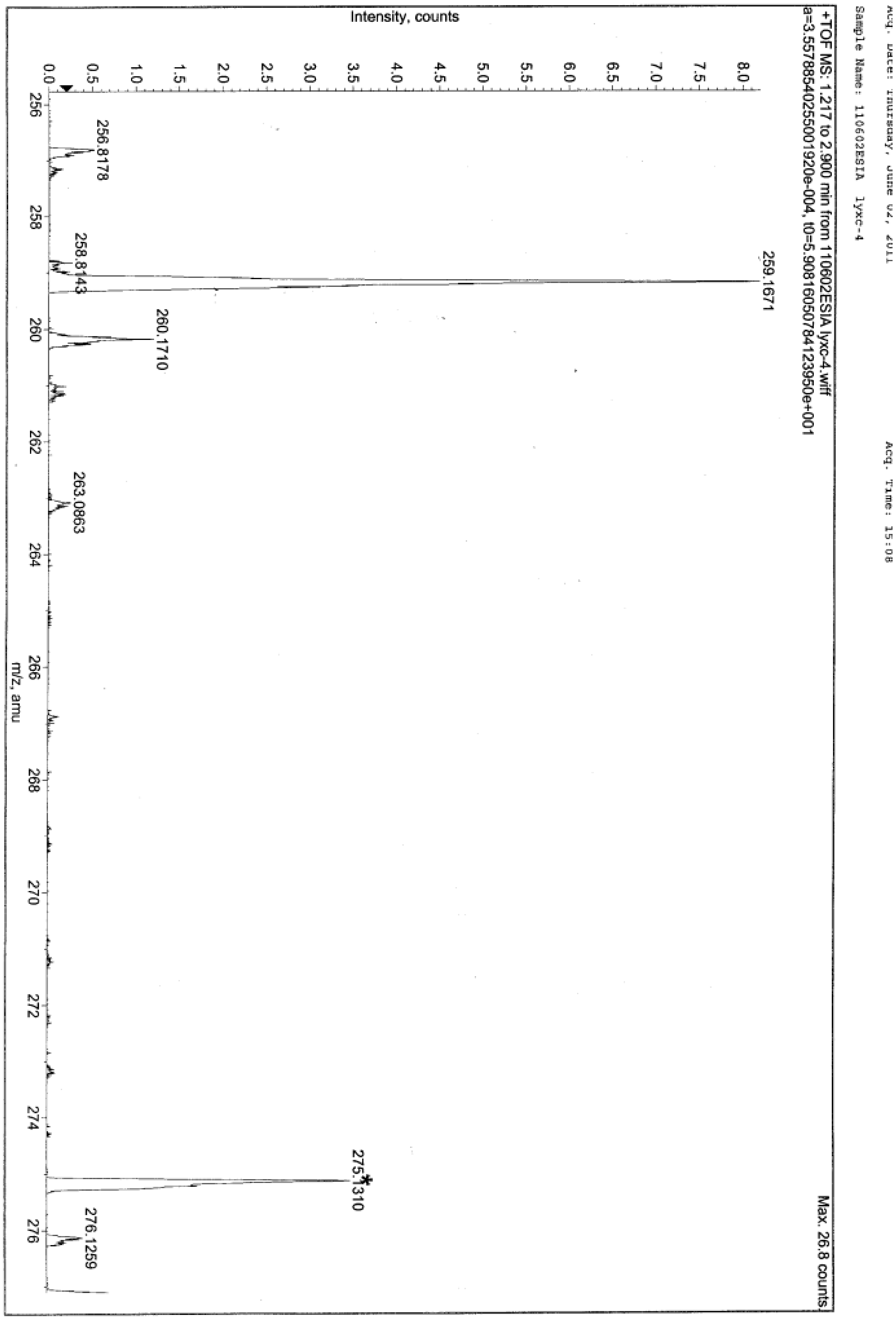

Figure 16S. $^{1}$H NMR of compound **3**.

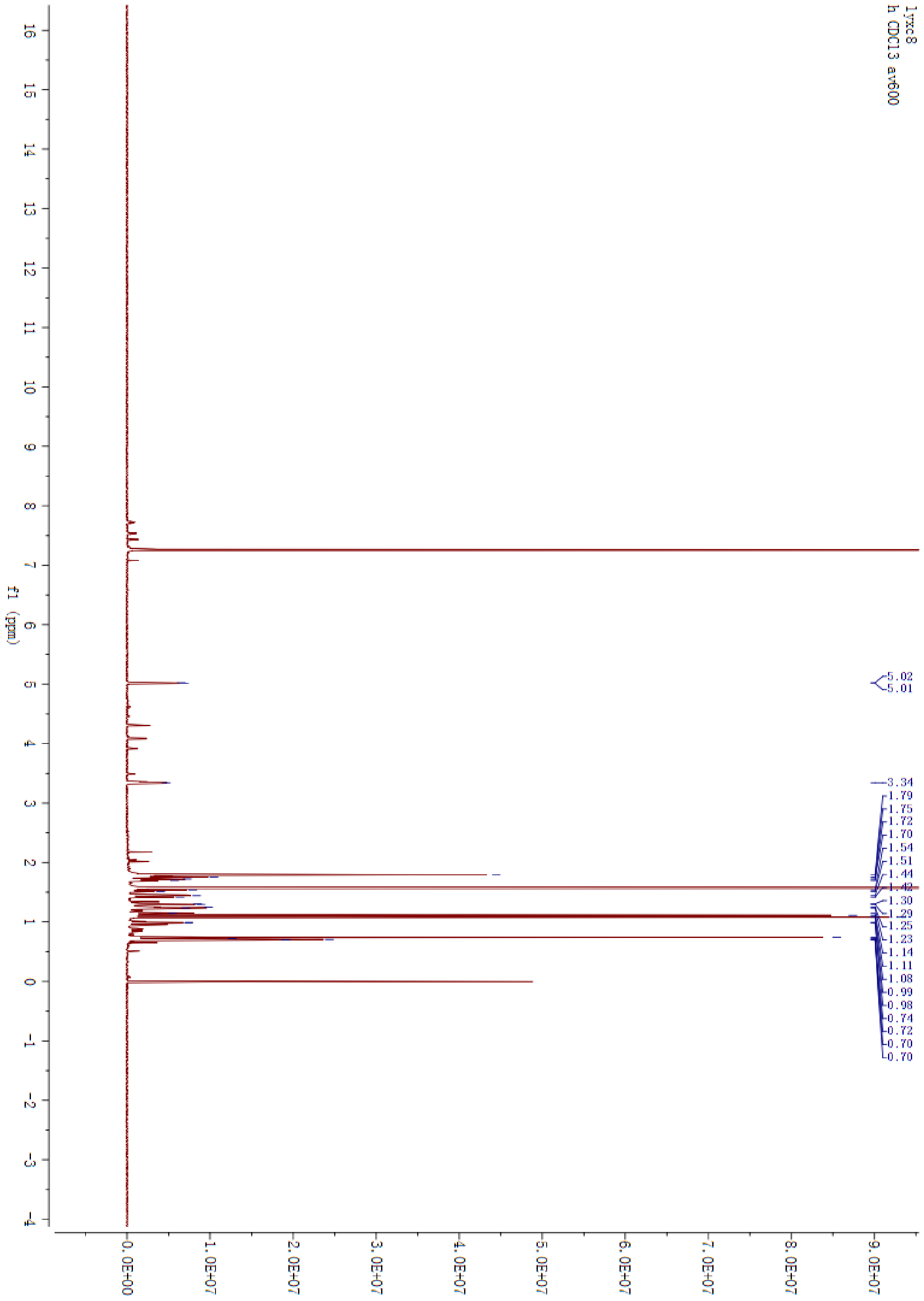

Figure 17S. $^{13}$C NMR and DEPT of compound **3**.

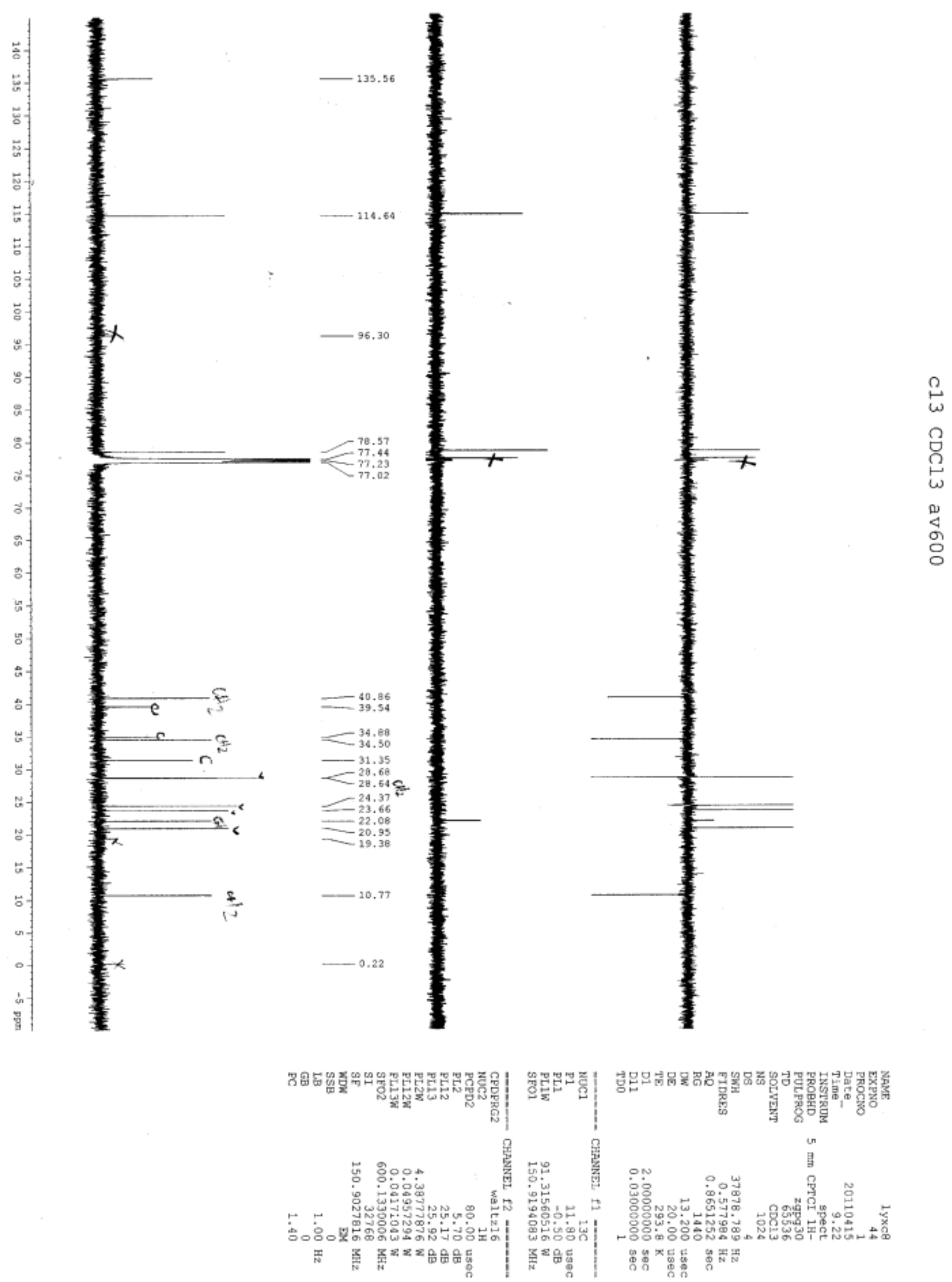

Figure 18S. HSQC of compound **3**.

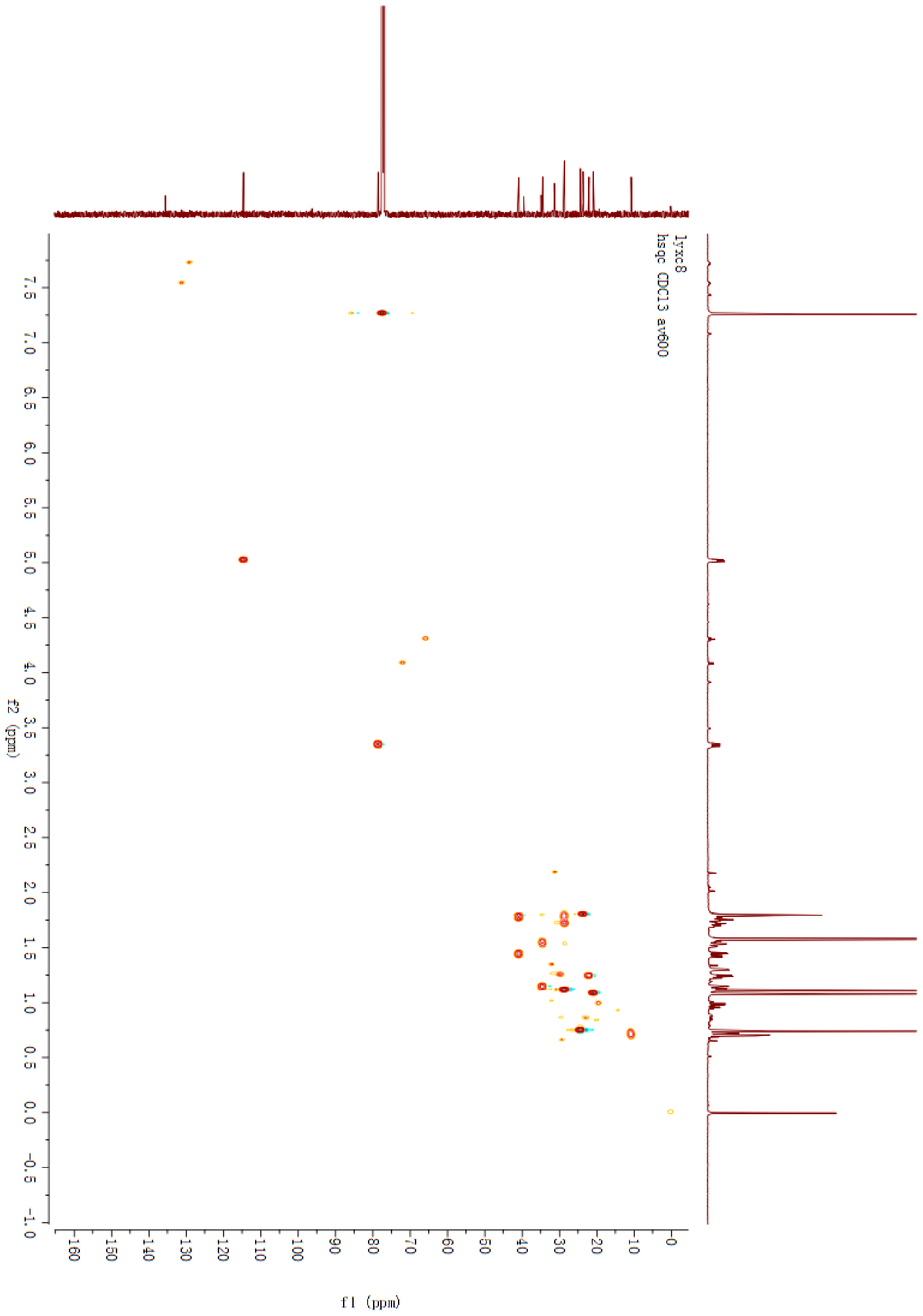

Figure 19S. HMBC of compound **3**.

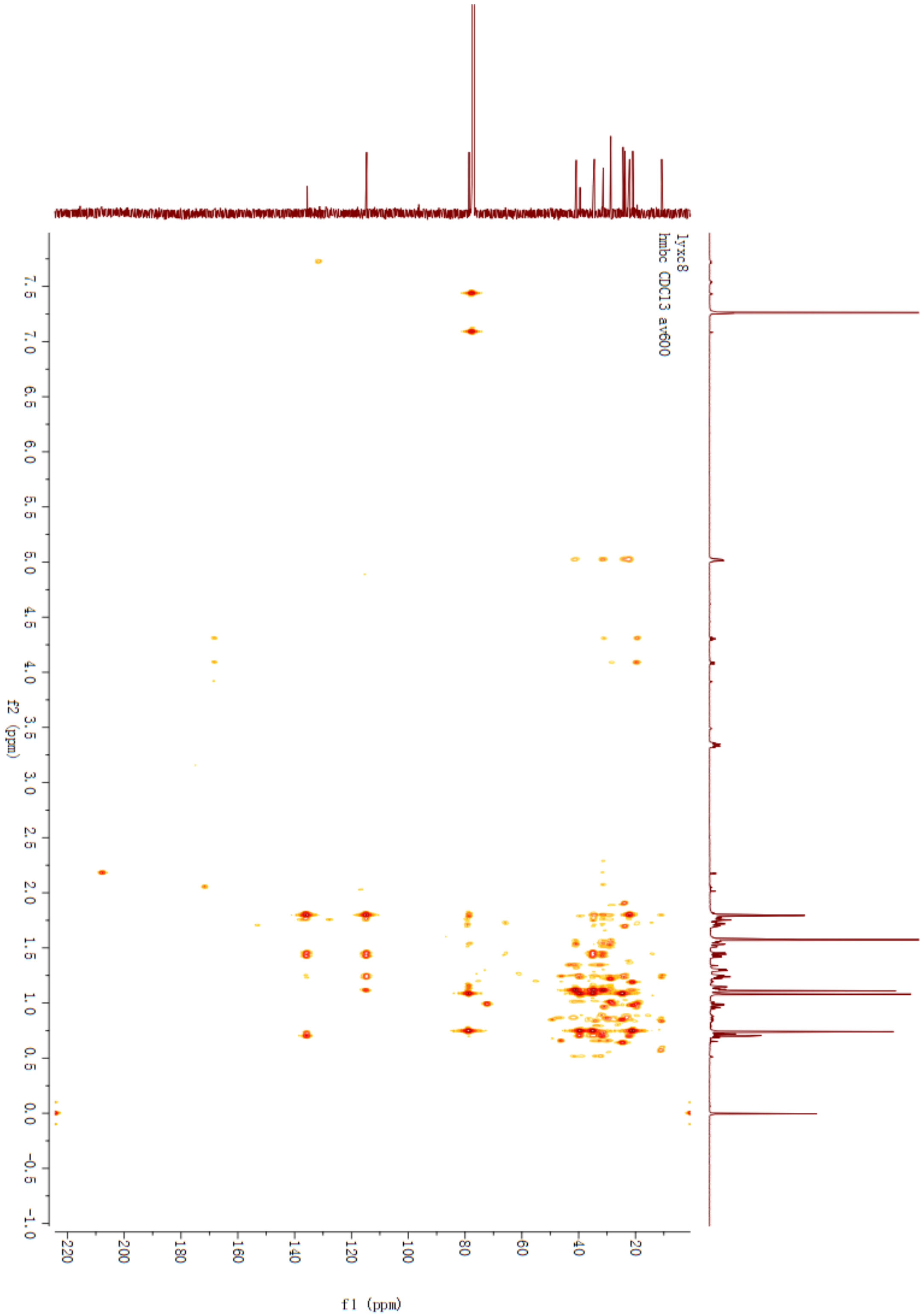

Figure 20S. $^{1}H$-$^{1}H$ COSY of compound **3**.

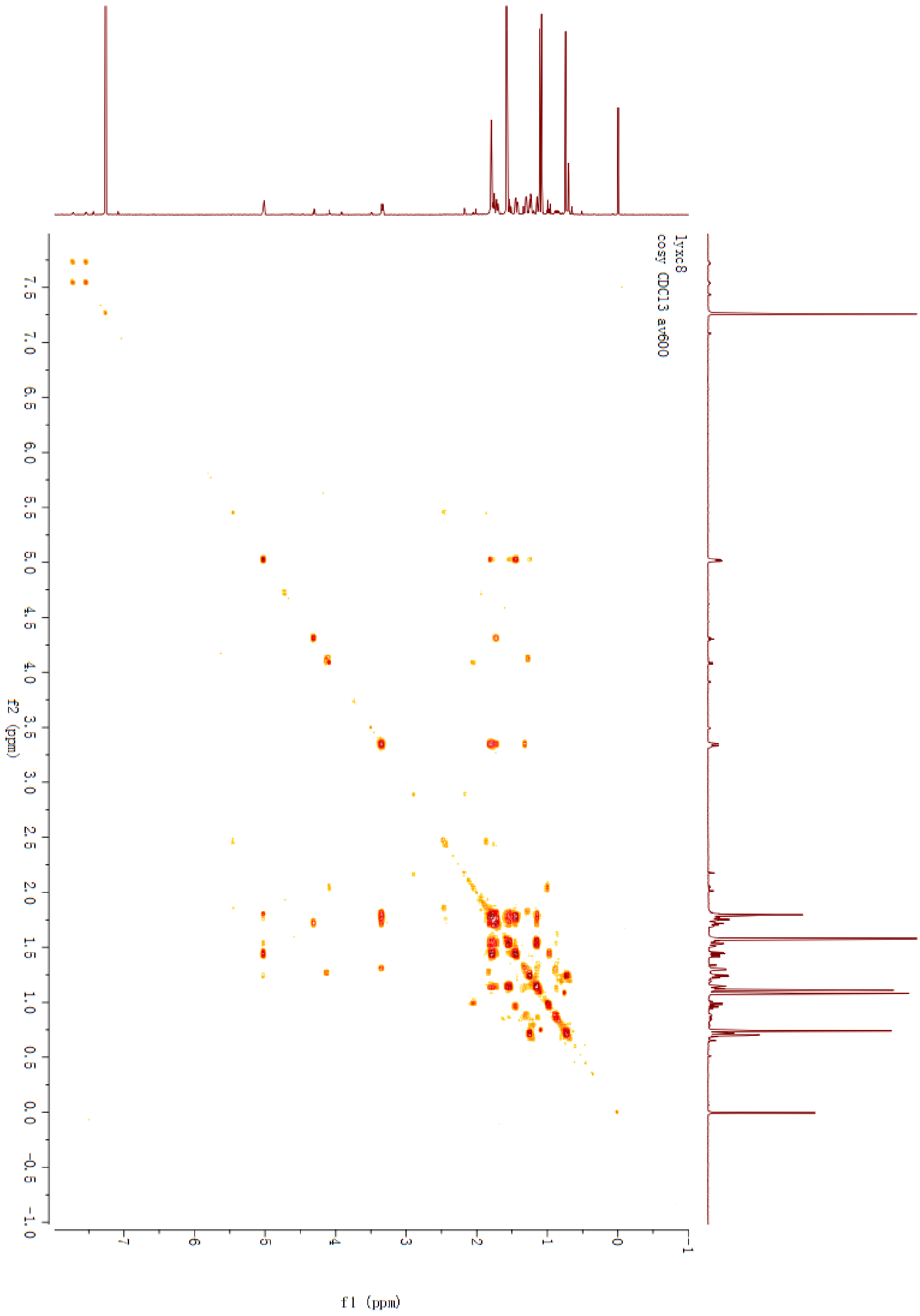

Figure 21S. ROESY of compound **3**.

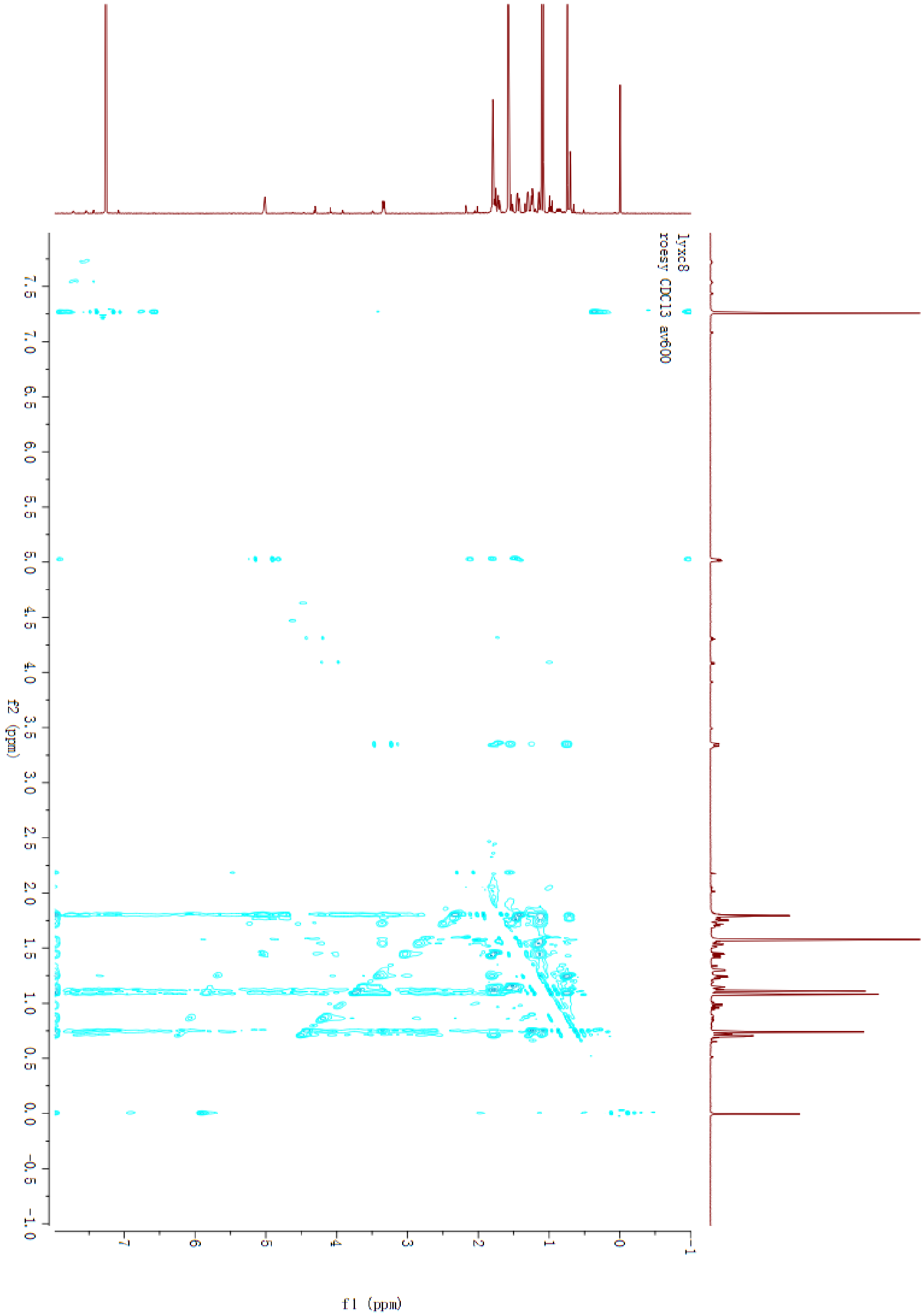

Figure 22S. HRESIMS of compound **3**.

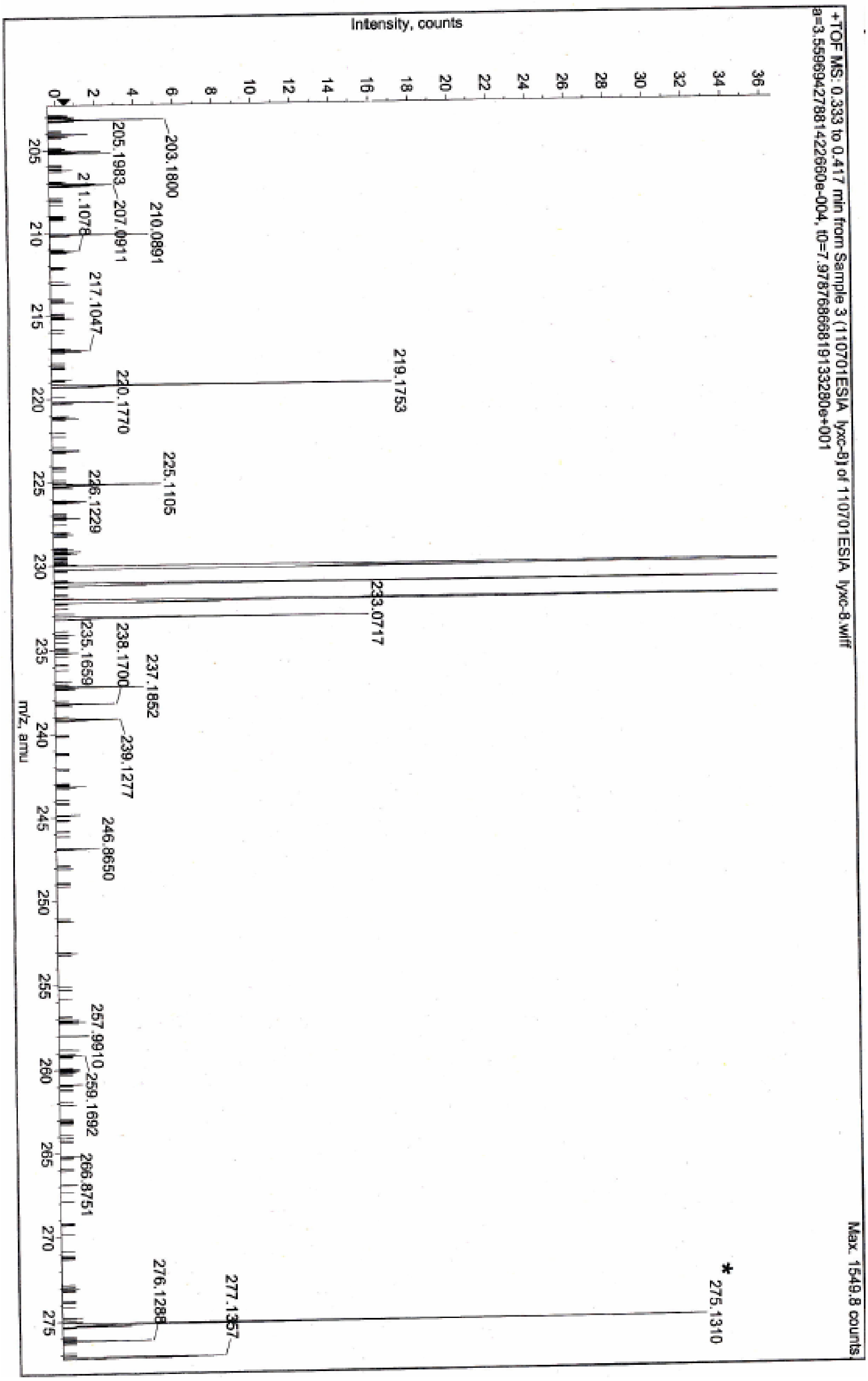

Figure 23S. $^{1}H$ NMR of compound **4**.

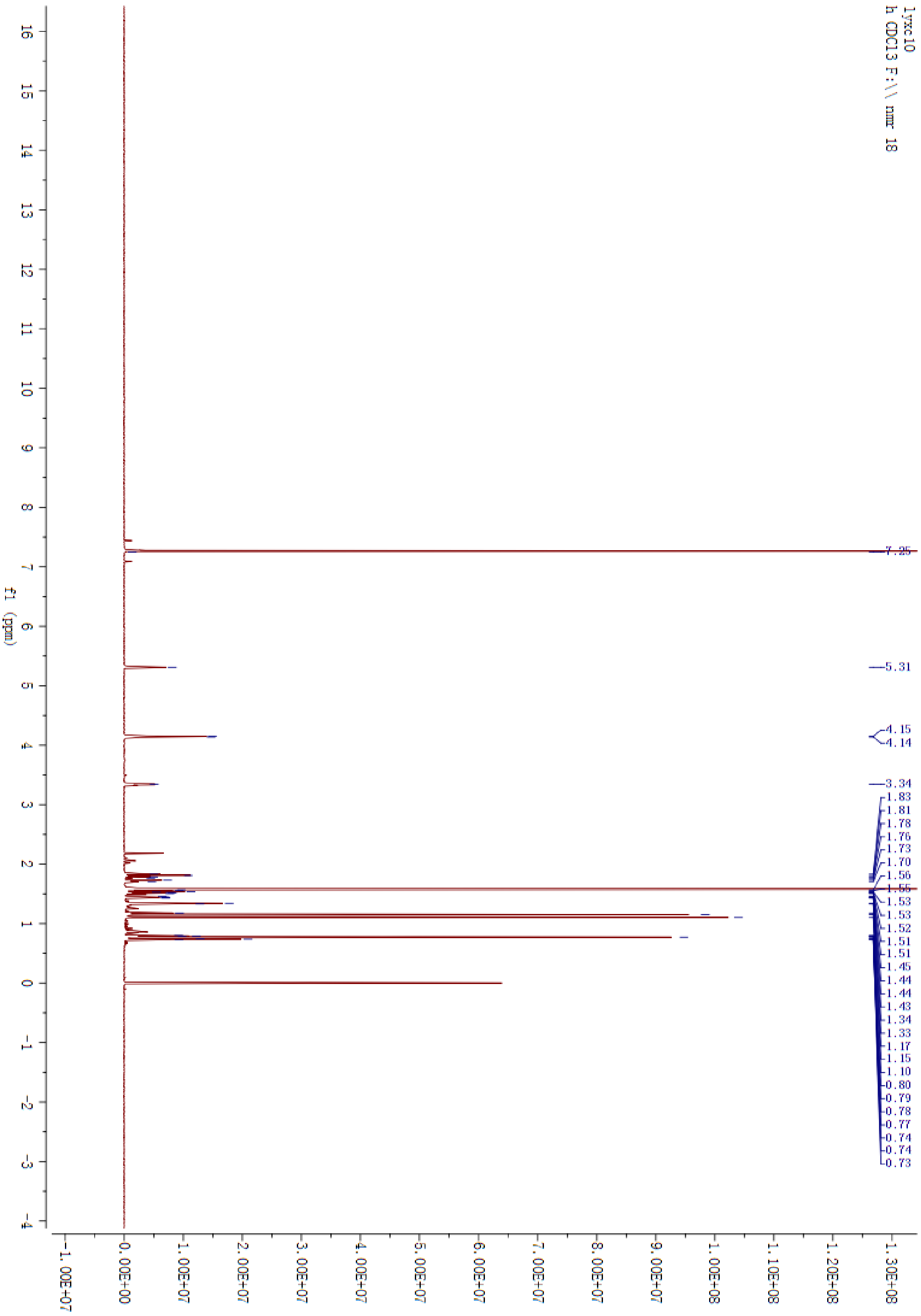

Figure 24S. $^{13}C$ NMR and DEPT of compound **4**.

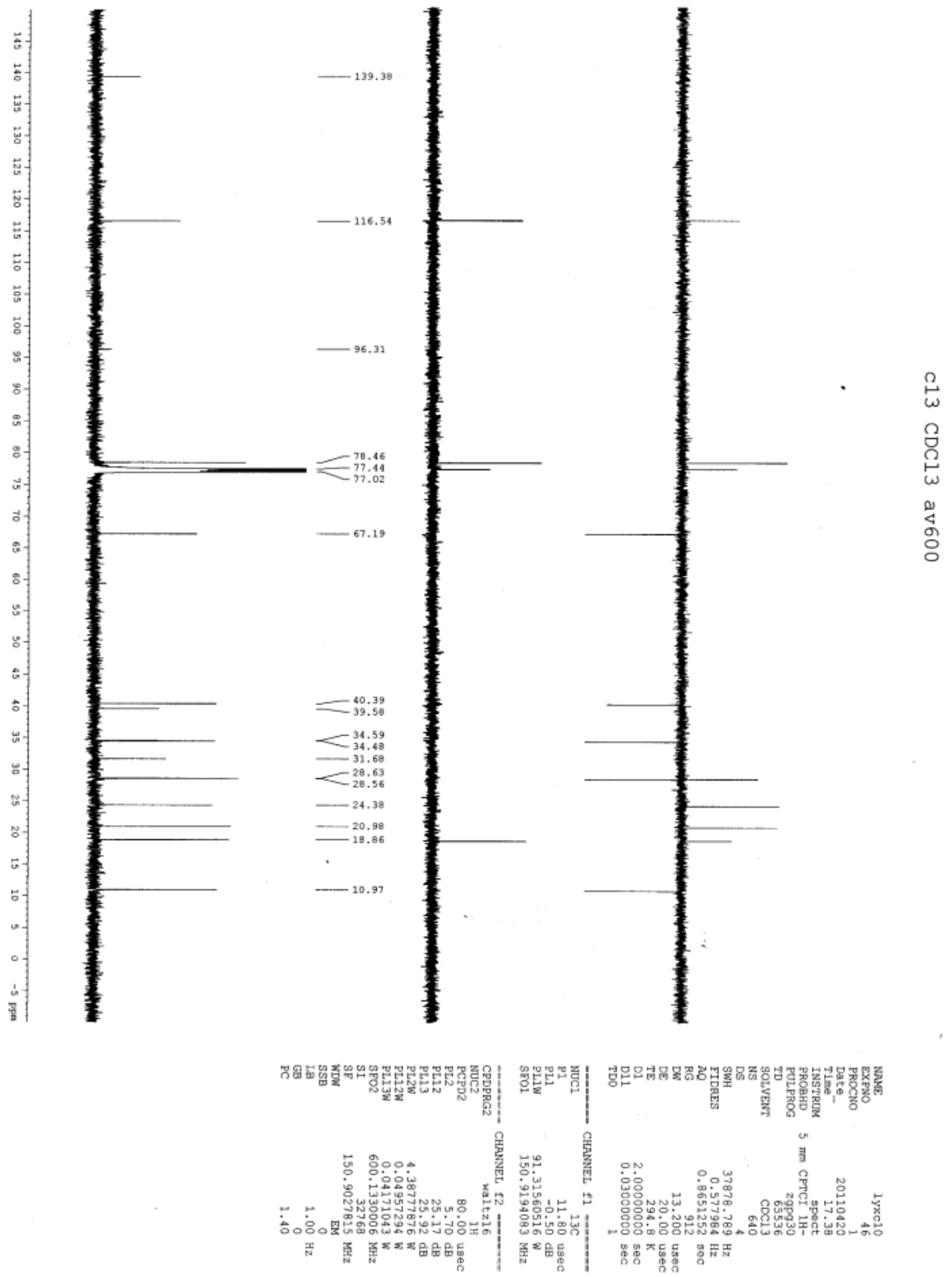

Figure 25S. HSQC of compound **4**.

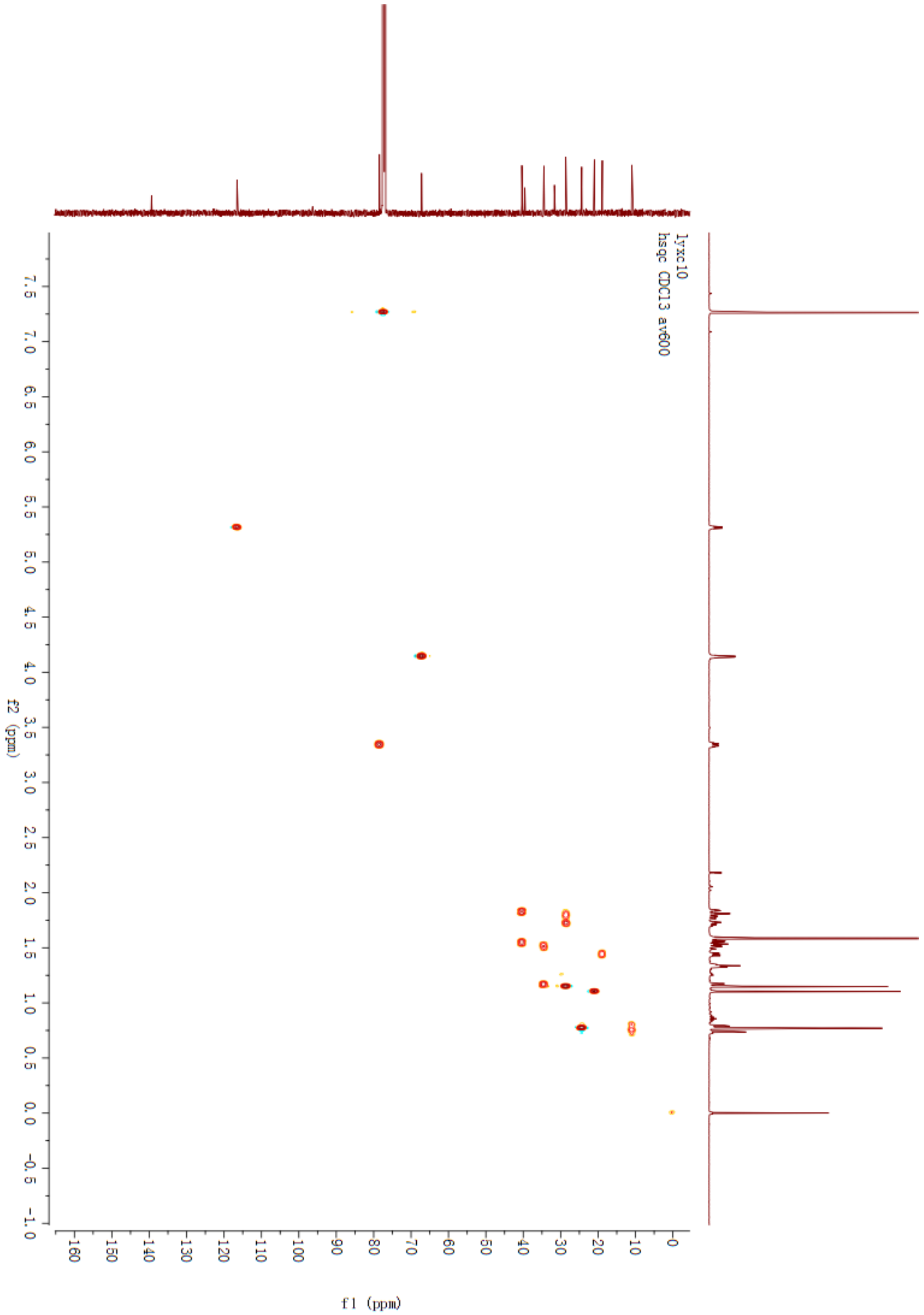

Figure 26S. HMBC of compound **4**.

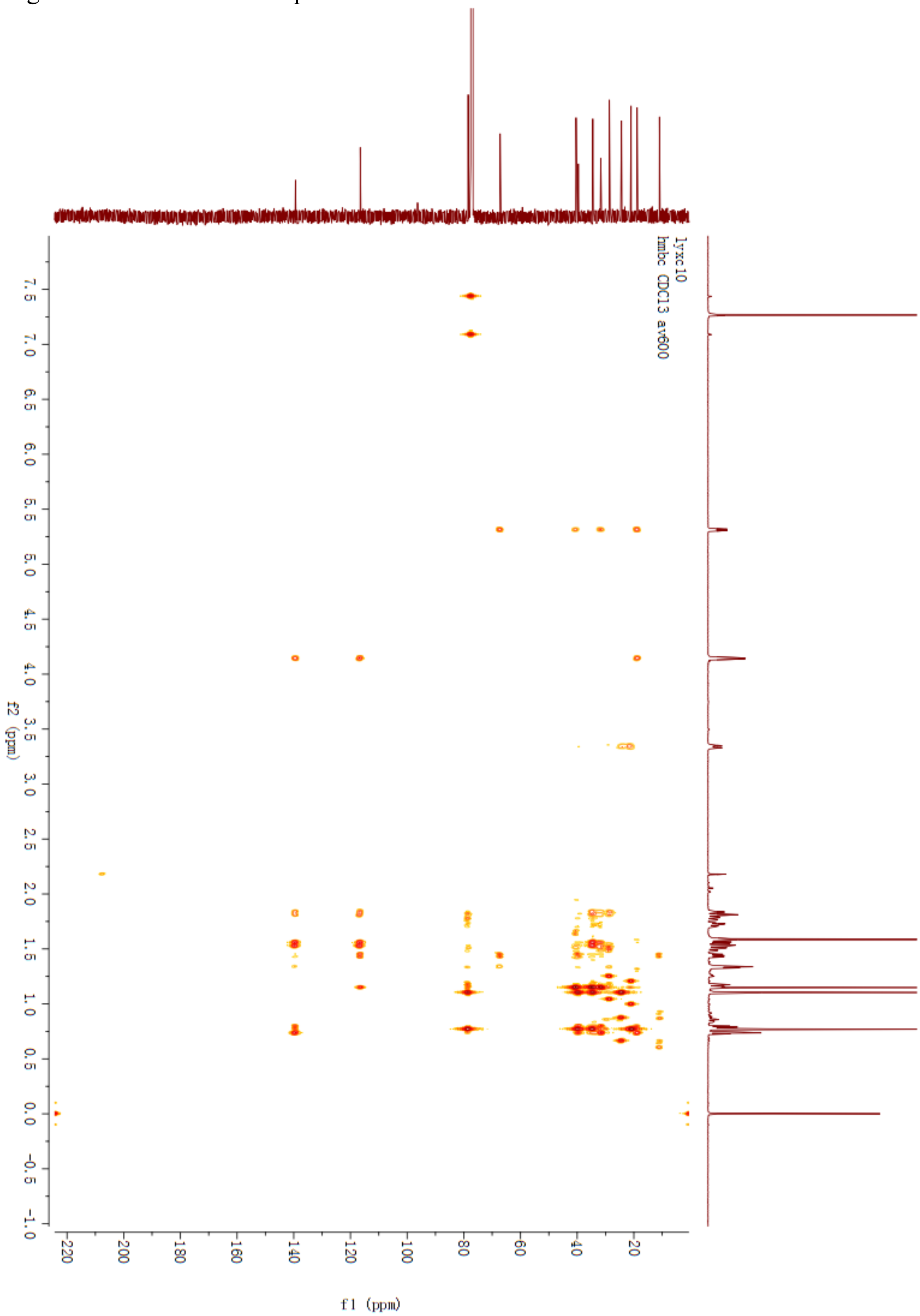

Figure 27S. $^{1}H$-$^{1}H$ COSY of compound **4**.

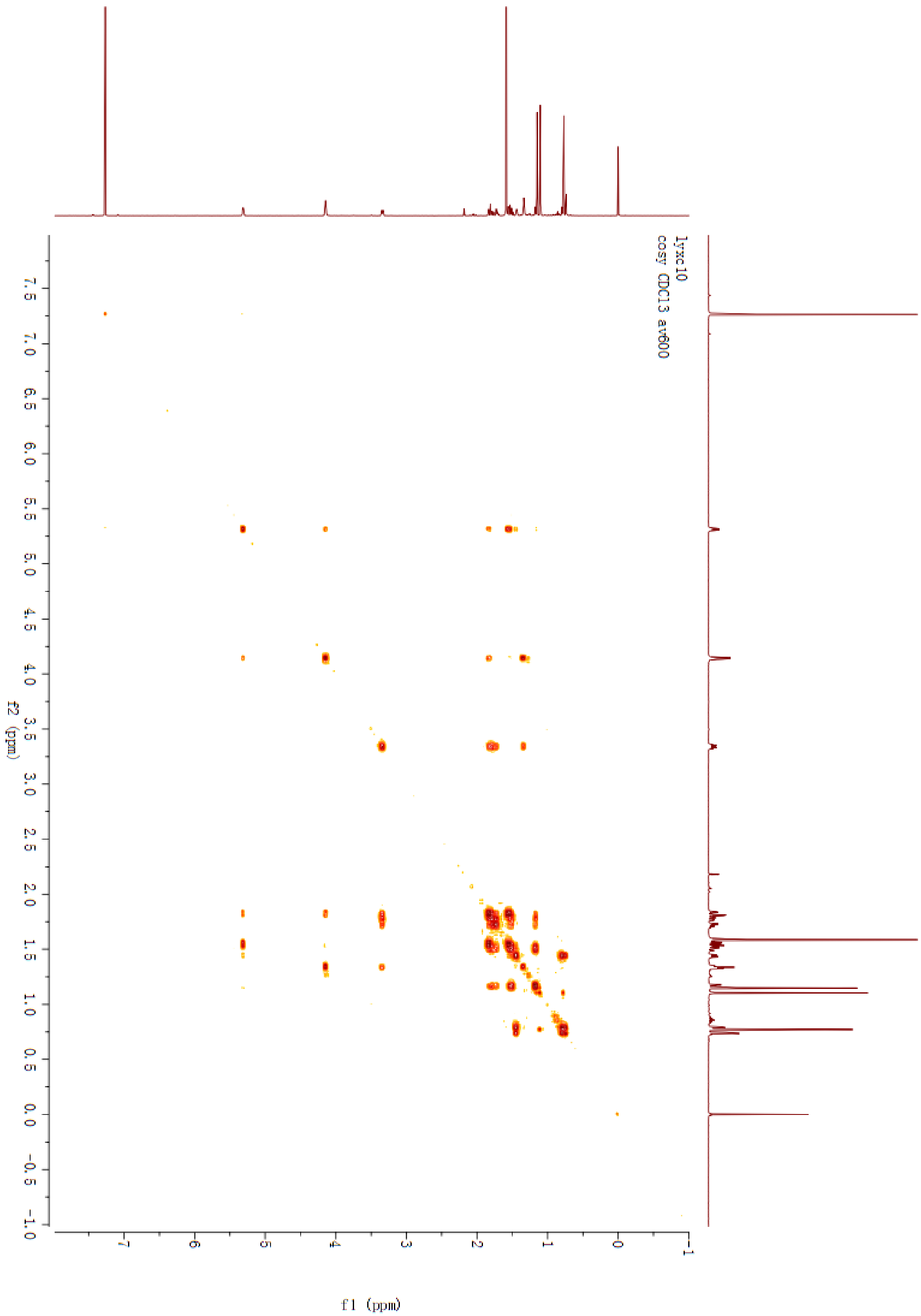

Figure 28S. ROESY of compound **4**.

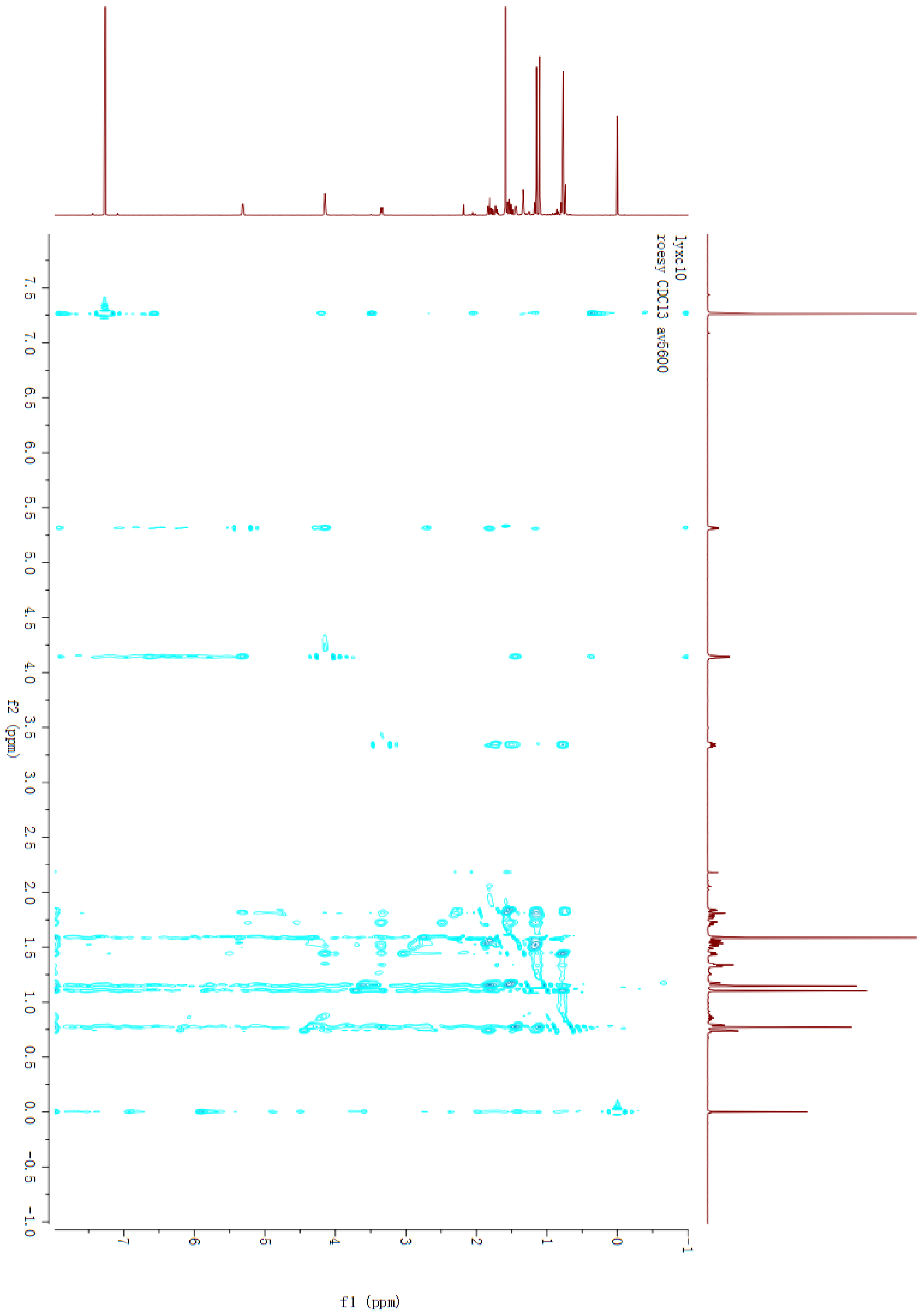

Figure 29S. HRESIMS of compound **4**.

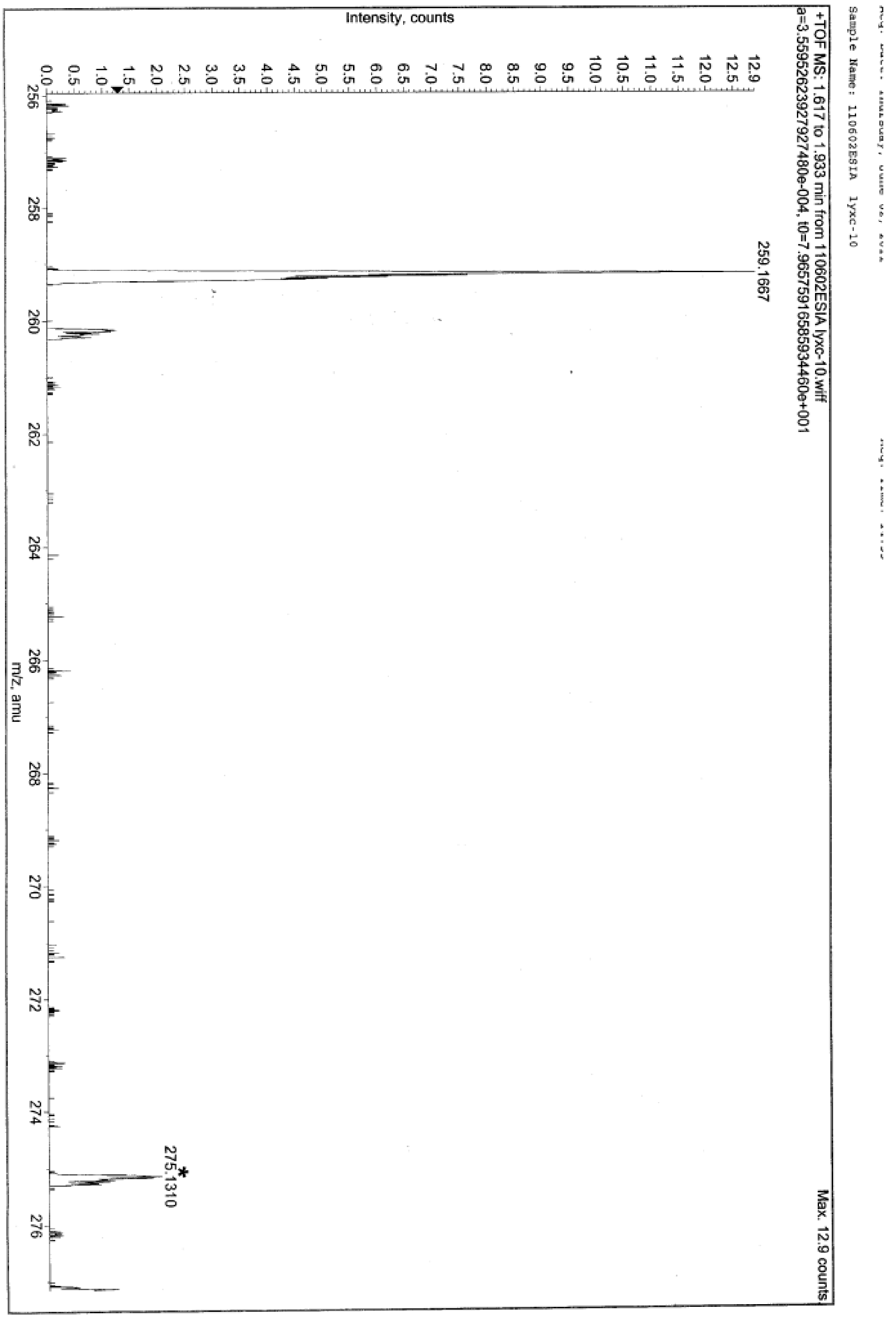

Figure 30S. $^{1}$H NMR of compound **5**.

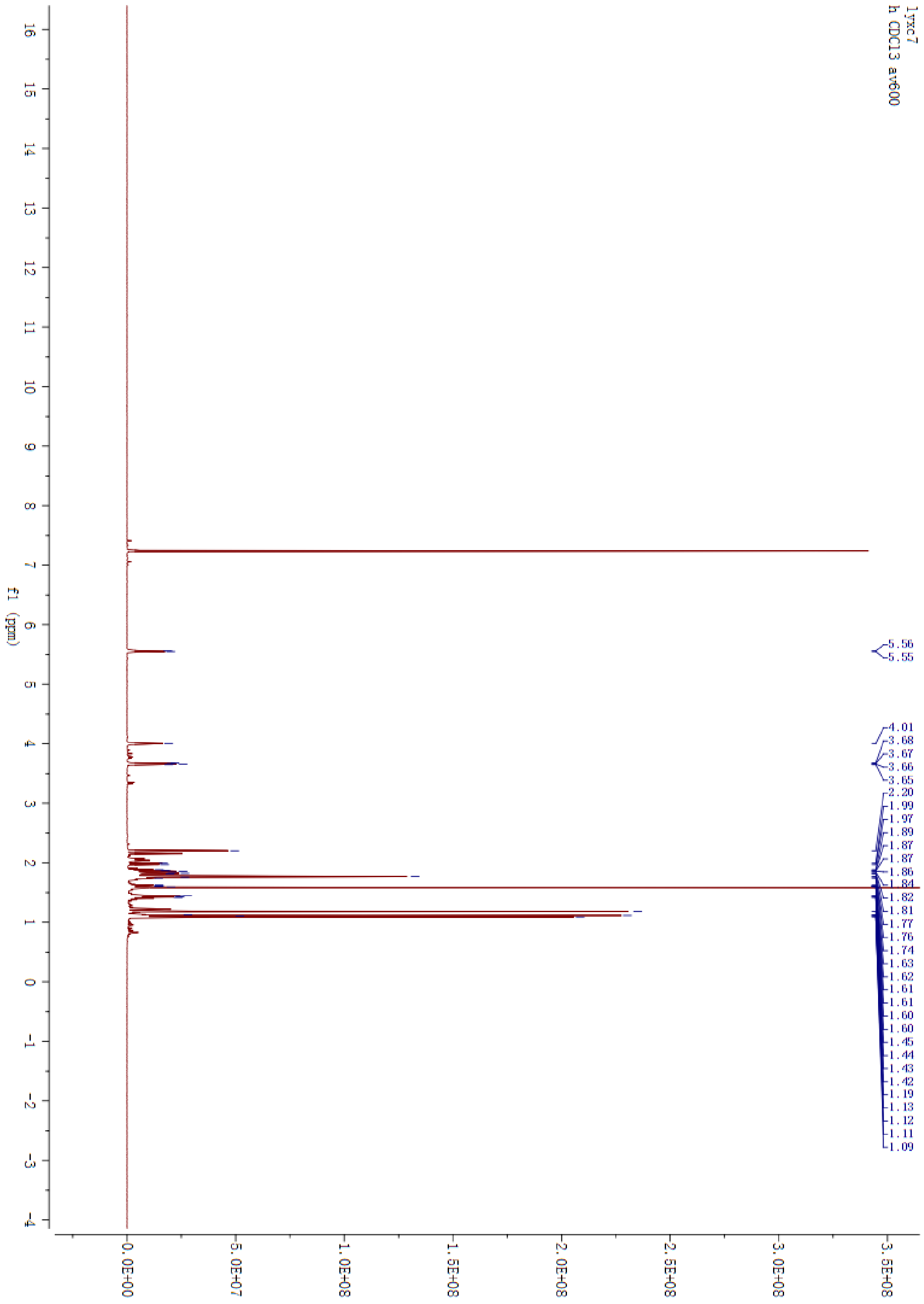

Figure 31S. $^{13}$C NMR and DEPT of compound **5**.

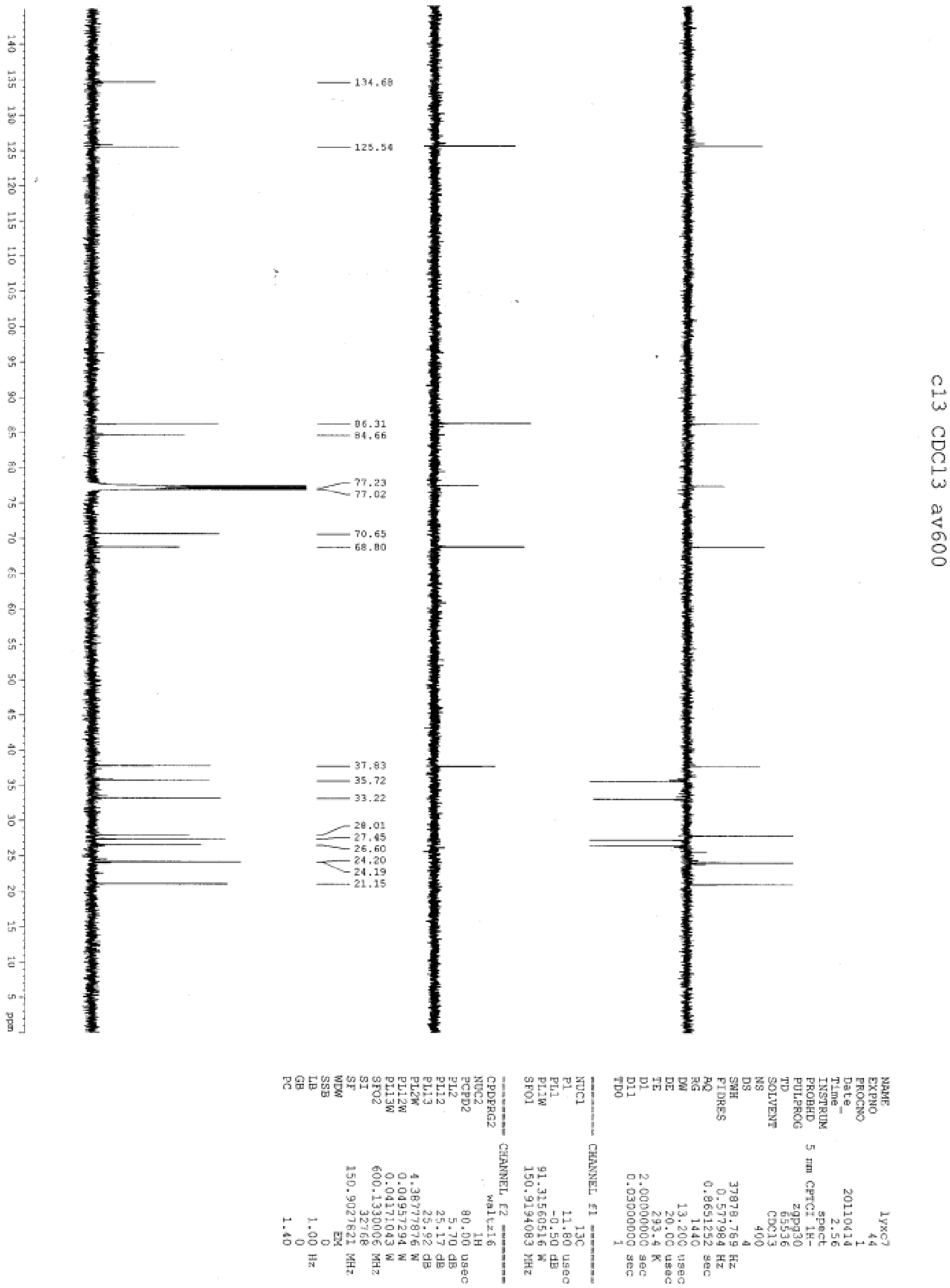

Figure 32S. HSQC of compound **5**.

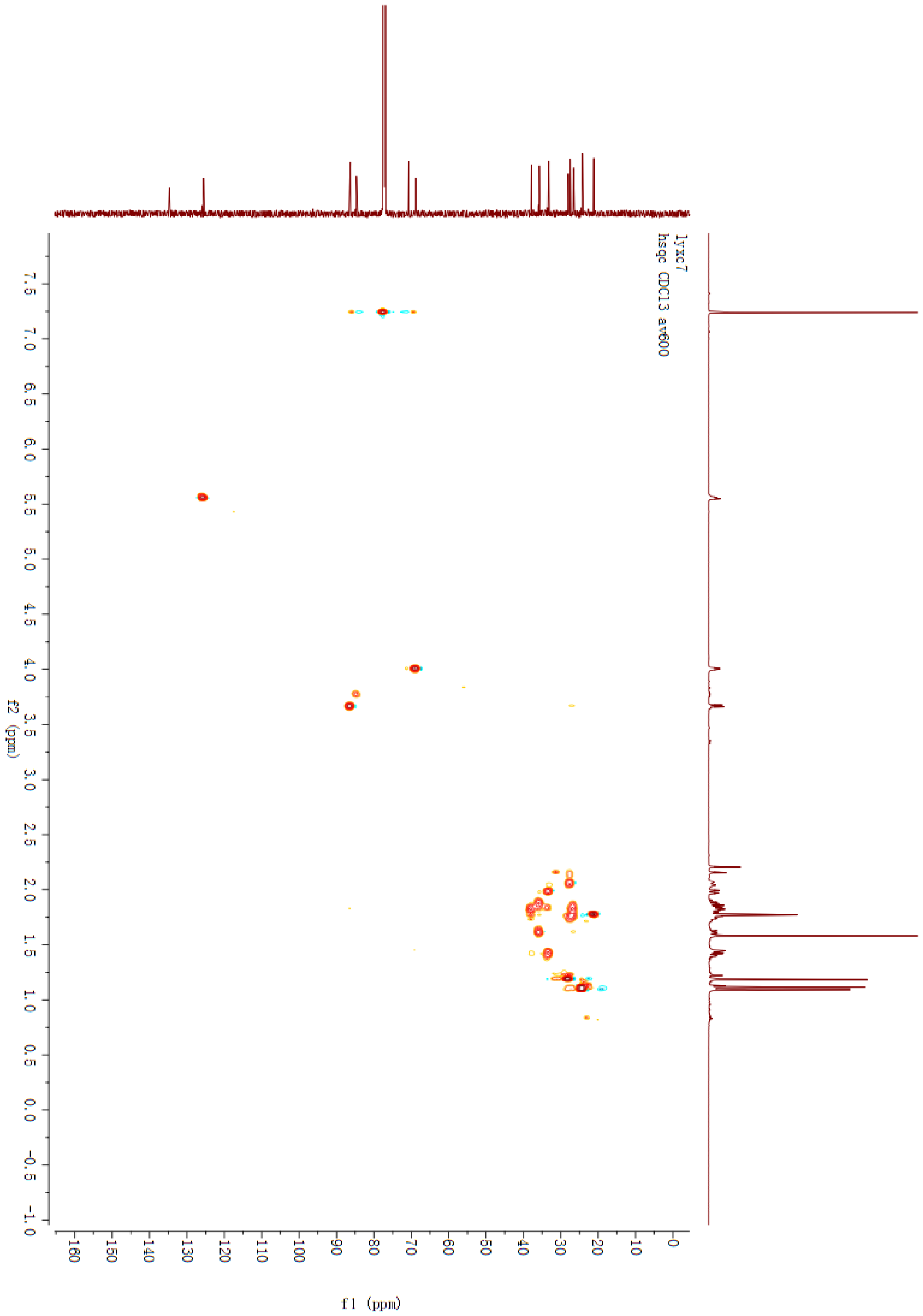

Figure 33S. HMBC of compound **5**.

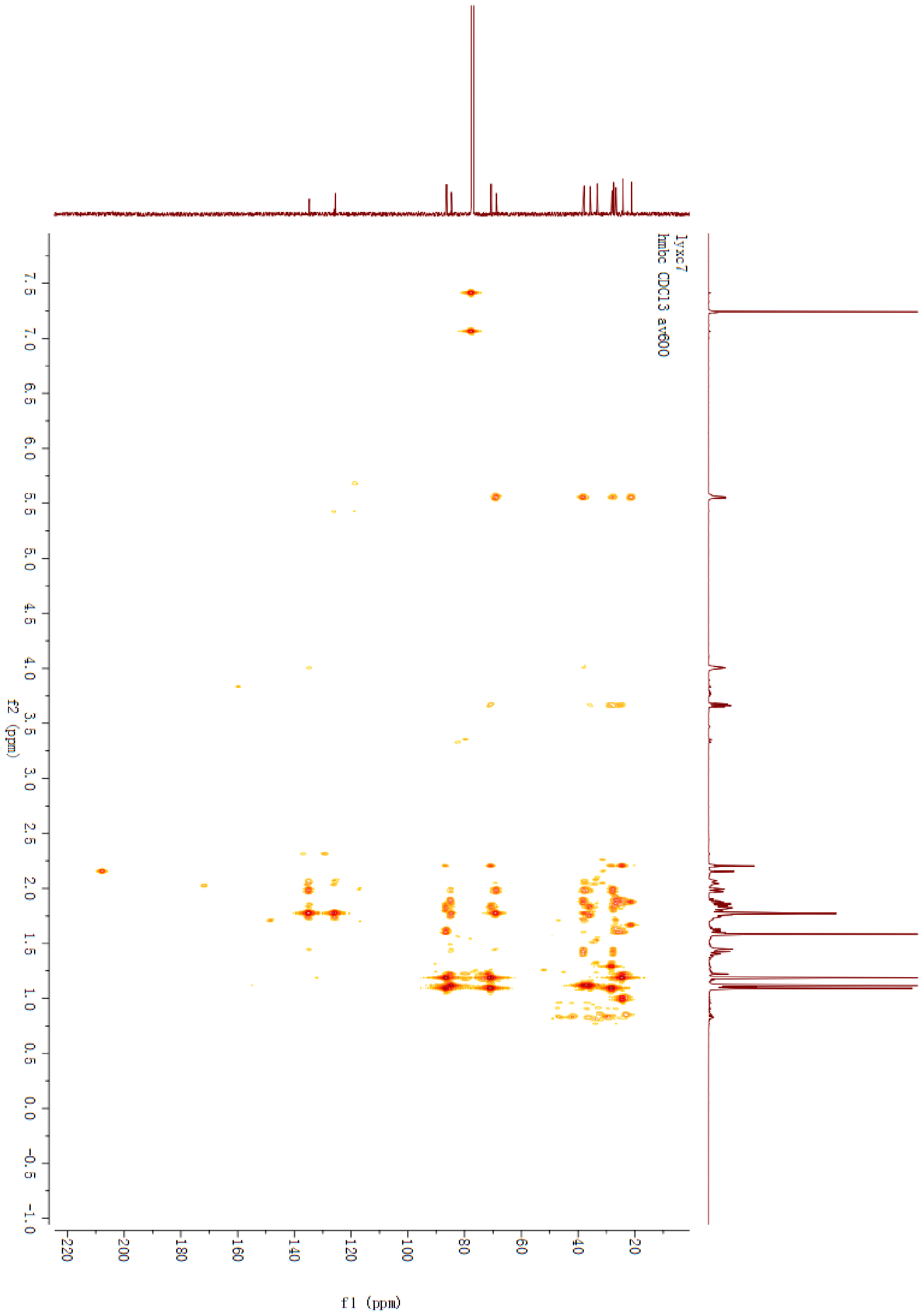

Figure 34S. $^1H$-$^1H$ COSY of compound **5**.

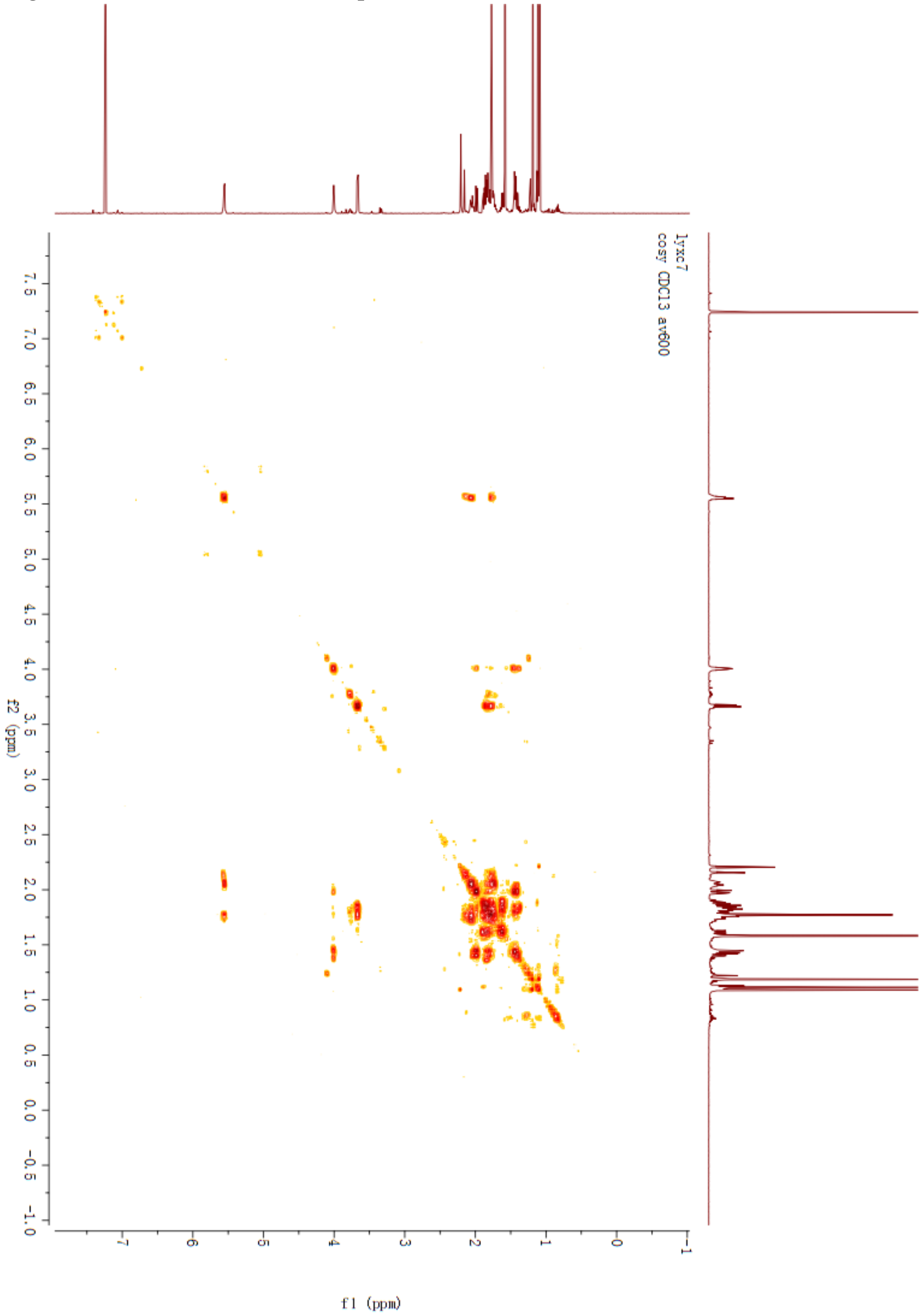

Figure 35S. ROESY of compound **5**.

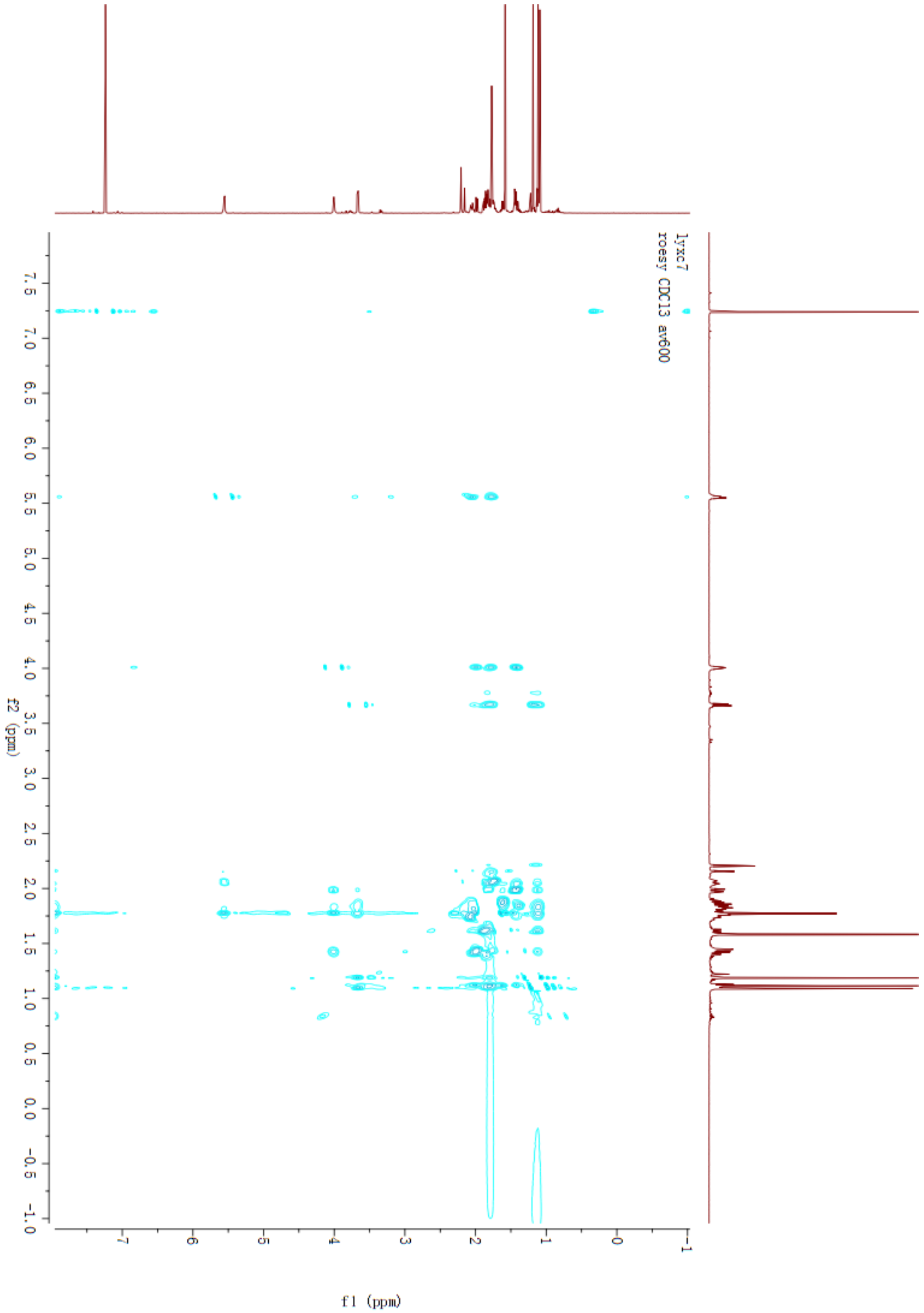

Figure 36S. HRESIMS of compound **5**.

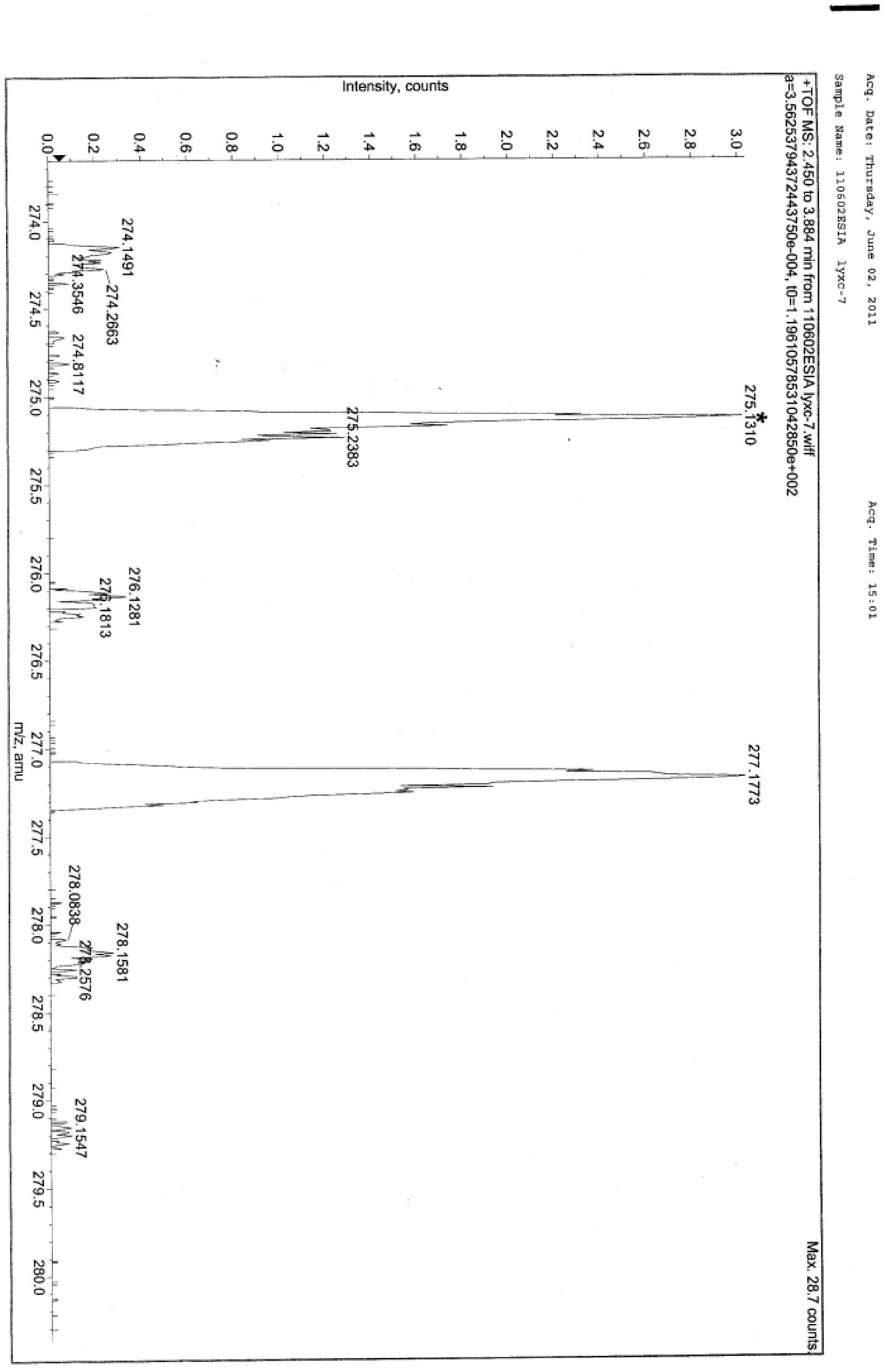

Figure 37S. $^{1}$H NMR of compound **6**.

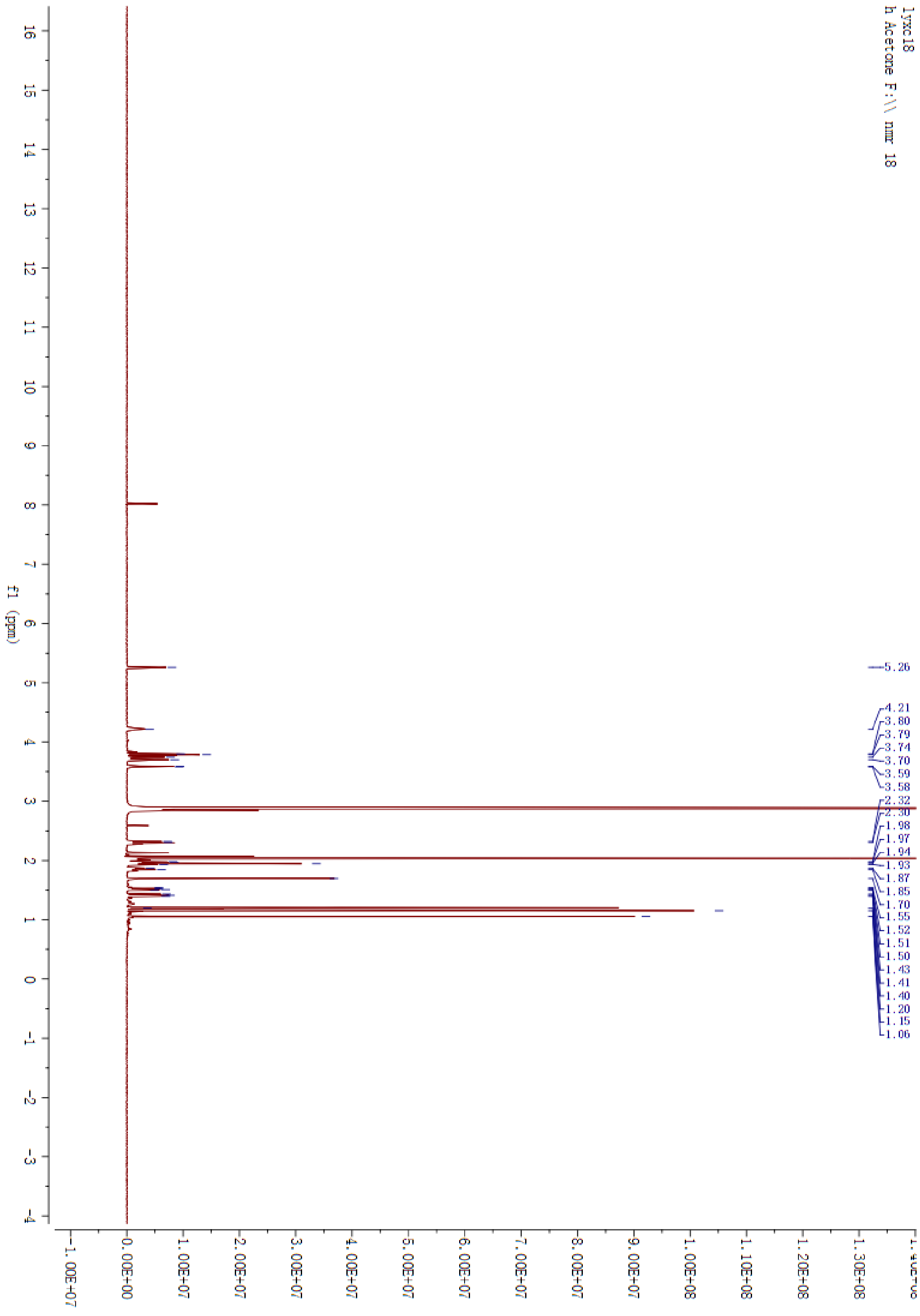

Figure 38S. $^{13}$C NMR and DEPT of compound **6**.

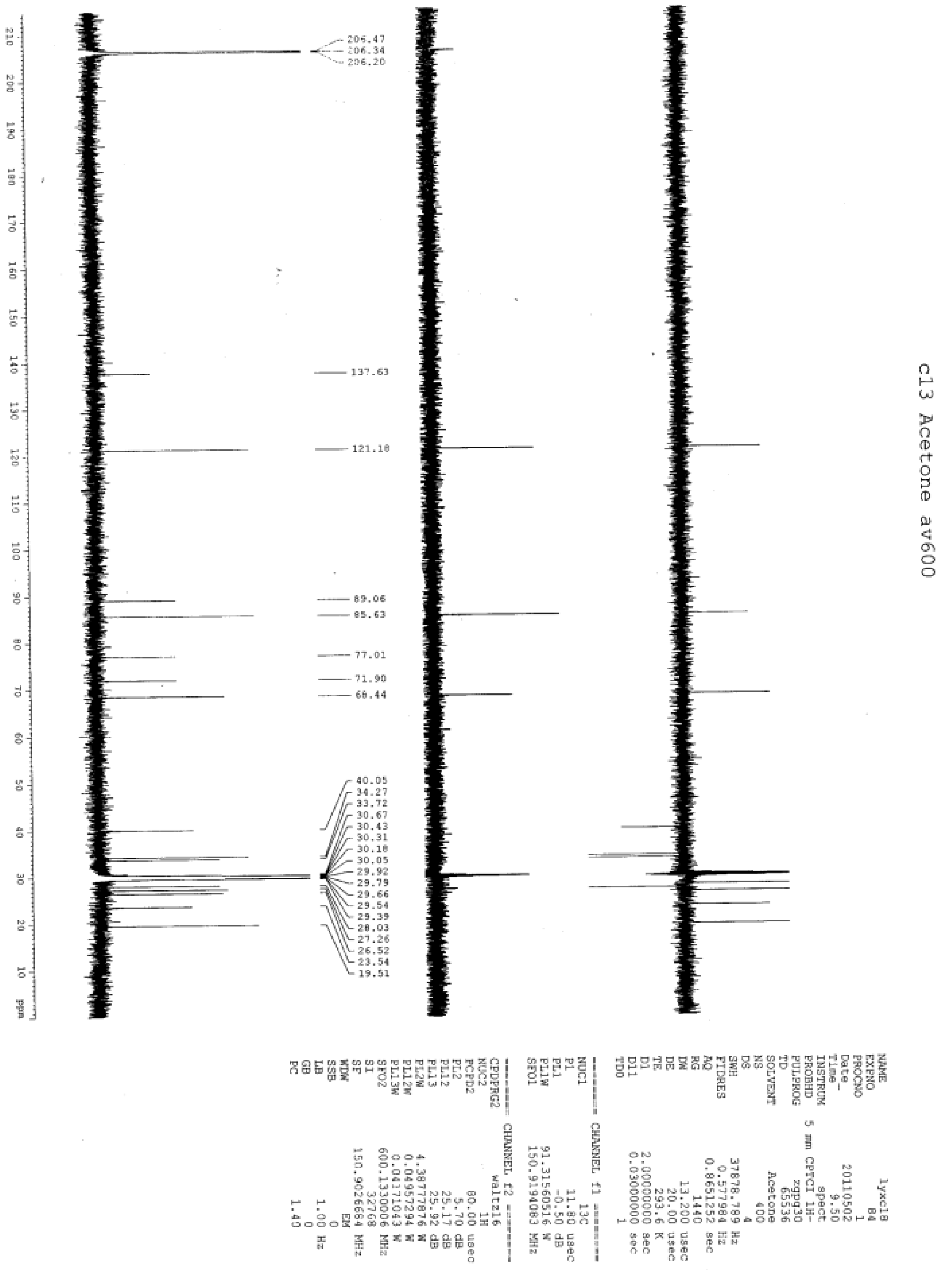

Figure 39S. HSQC of compound **6**.

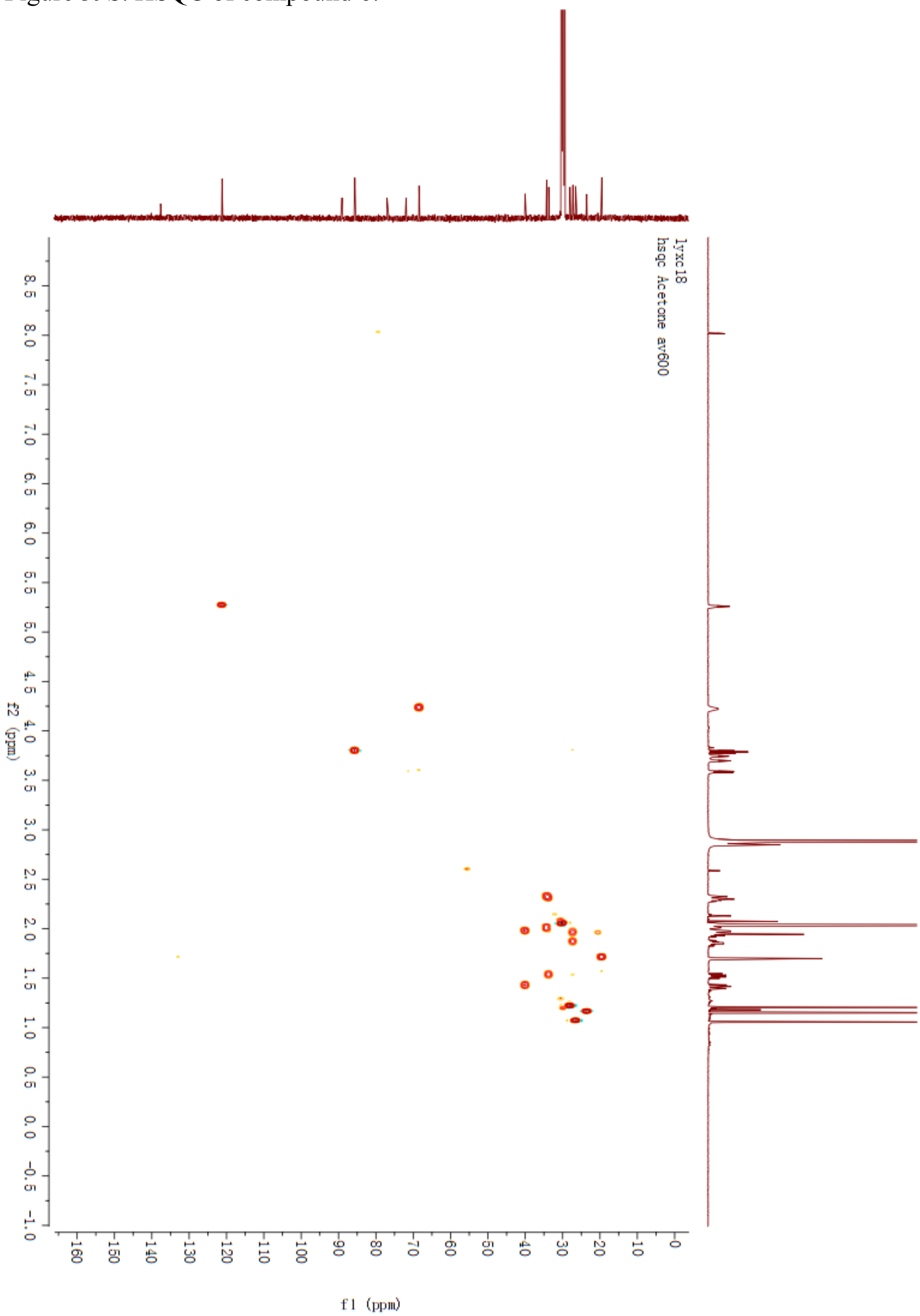

Figure 40S. HMBC of compound **6**.

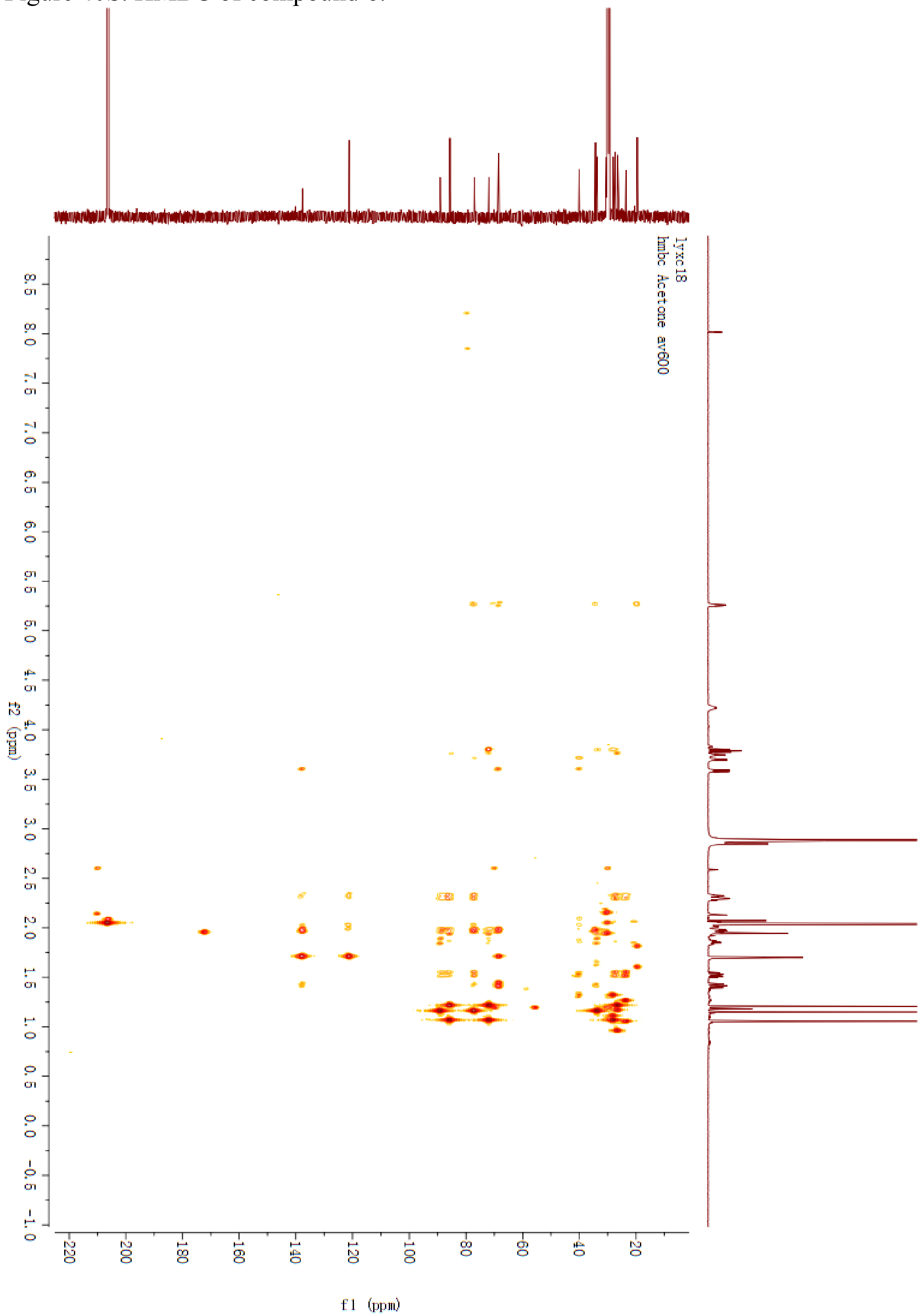

Figure 41S. HRESIMS of compound **6**.

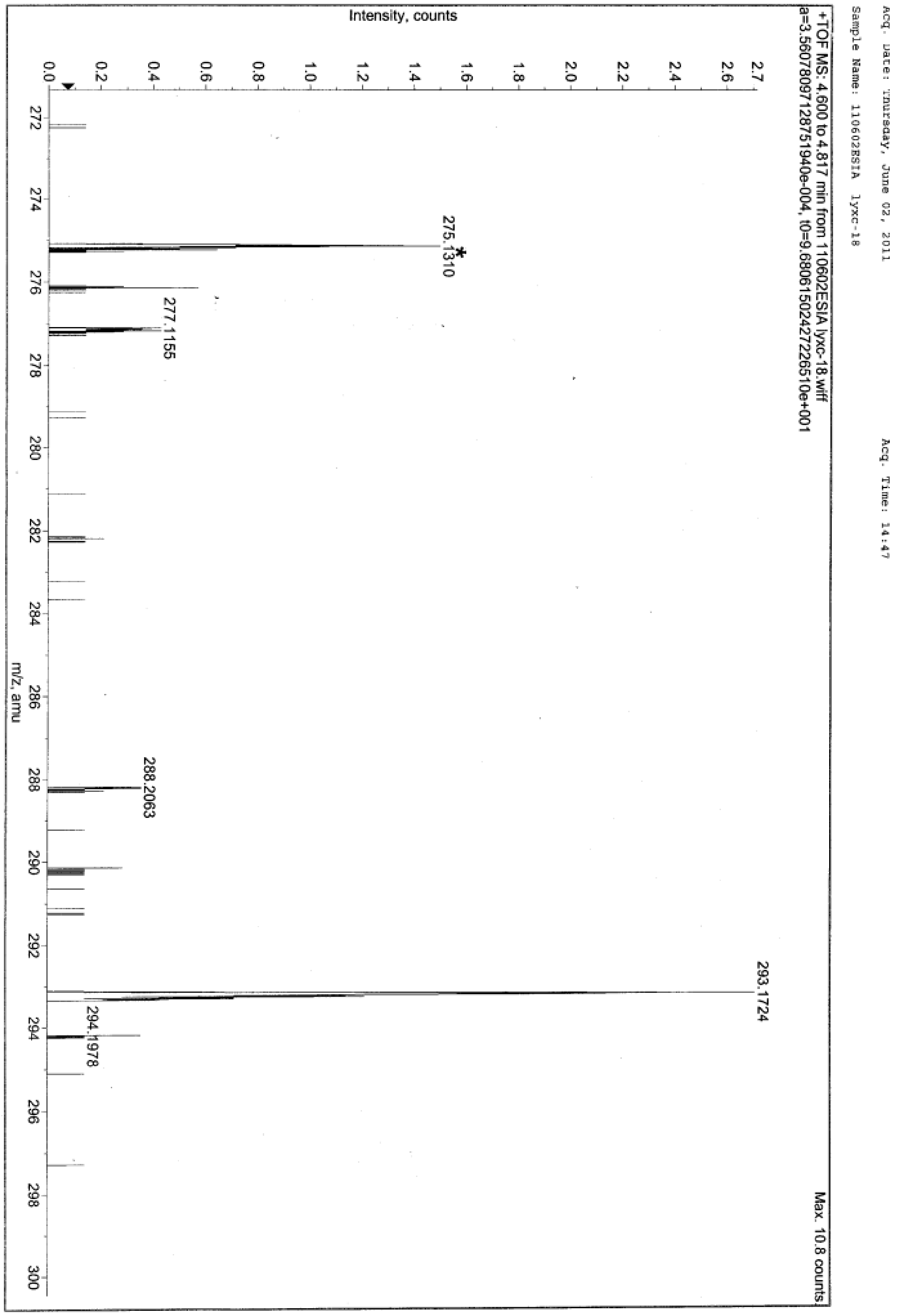

Figure 42S. $^{1}$H NMR of compound **7**.

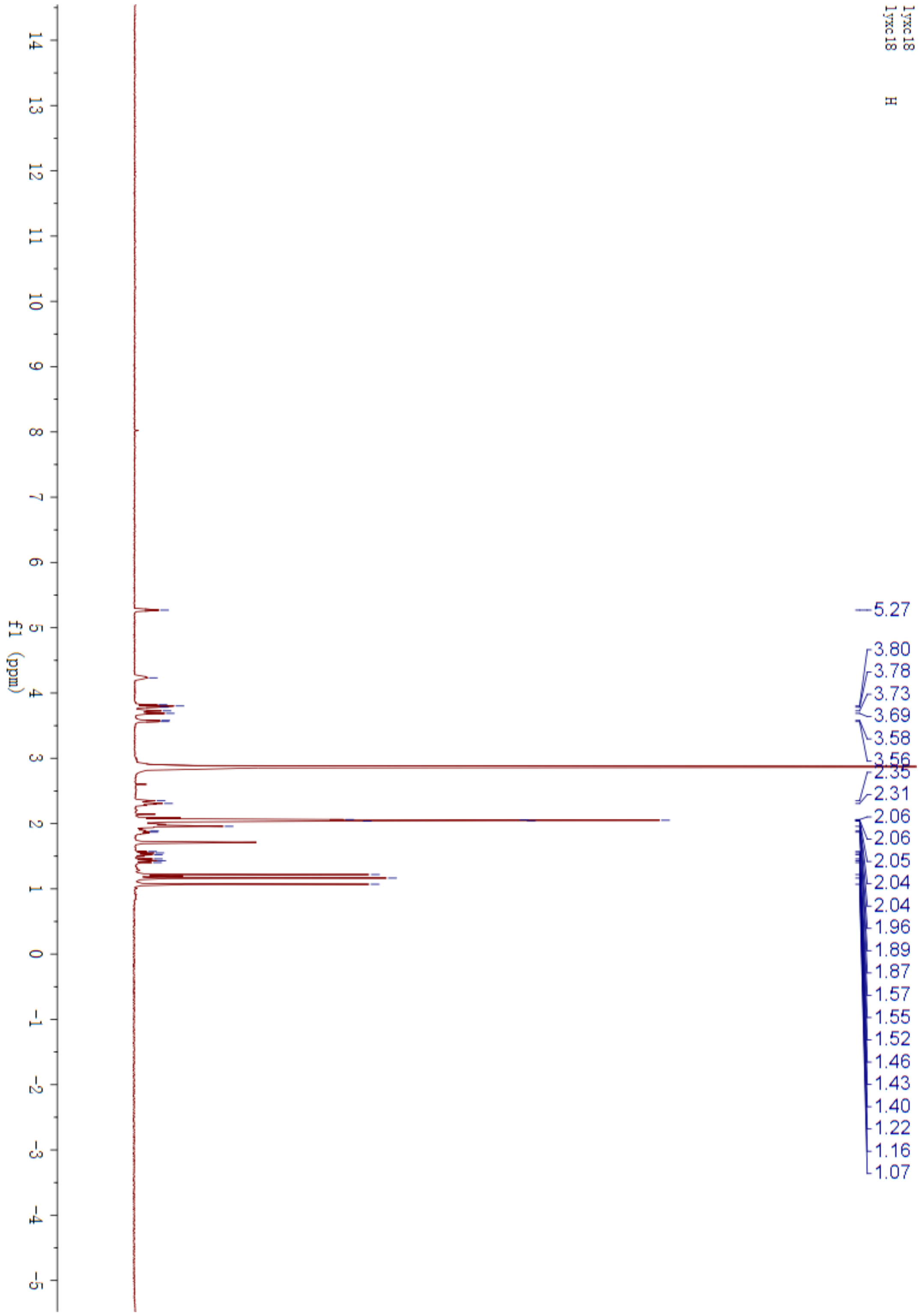

Figure 43S. $^{13}C$ NMR of compound **7**.

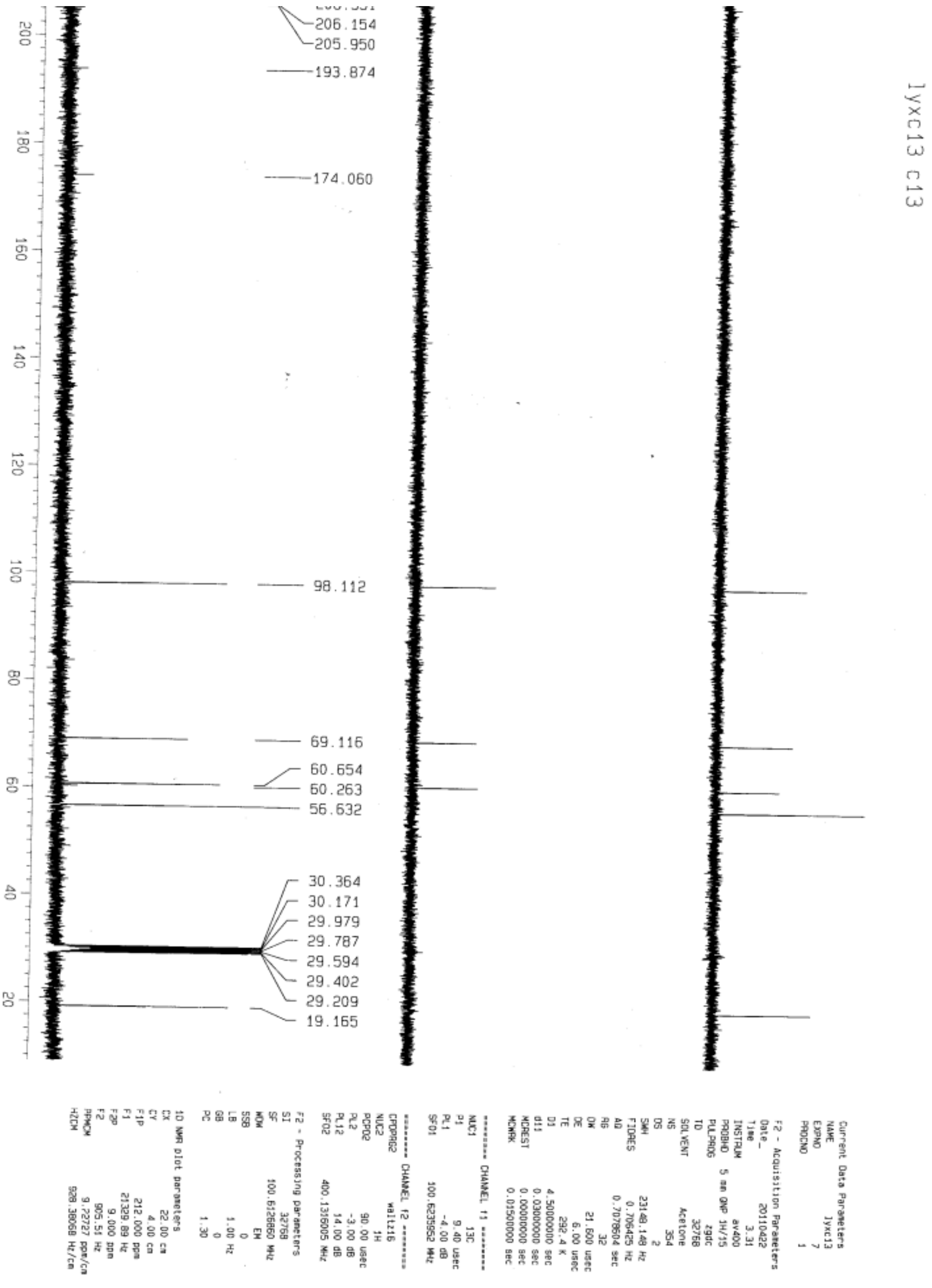

Supplement: Supplementary file 1 — Supplementary material, approximately 2.39 MB. [file 13659_2011_11_MOESM1_ESM.pdf]
